# Dynamic phase separation of the androgen receptor and its coactivators key to regulate gene expression

**DOI:** 10.1093/nar/gkac1158

**Published:** 2022-12-20

**Authors:** Fan Zhang, Maitree Biswas, Shabnam Massah, Joseph Lee, Shreyas Lingadahalli, Samantha Wong, Christopher Wells, Jane Foo, Nabeel Khan, Helene Morin, Neetu Saxena, Sonia H Y Kung, Bei Sun, Ana Karla Parra Nuñez, Christophe Sanchez, Novia Chan, Lauren Ung, Umut Berkay Altıntaş, Jennifer M Bui, Yuzhuo Wang, Ladan Fazli, Htoo Zarni Oo, Paul S Rennie, Nathan A Lack, Artem Cherkasov, Martin E Gleave, Jörg Gsponer, Nada Lallous

**Affiliations:** Vancouver Prostate Centre, Department of Urologic Sciences, University of British Columbia, 2660 Oak St., Vancouver, BC V6H 3Z6, Canada; Vancouver Prostate Centre, Department of Urologic Sciences, University of British Columbia, 2660 Oak St., Vancouver, BC V6H 3Z6, Canada; Vancouver Prostate Centre, Department of Urologic Sciences, University of British Columbia, 2660 Oak St., Vancouver, BC V6H 3Z6, Canada; Vancouver Prostate Centre, Department of Urologic Sciences, University of British Columbia, 2660 Oak St., Vancouver, BC V6H 3Z6, Canada; Vancouver Prostate Centre, Department of Urologic Sciences, University of British Columbia, 2660 Oak St., Vancouver, BC V6H 3Z6, Canada; Vancouver Prostate Centre, Department of Urologic Sciences, University of British Columbia, 2660 Oak St., Vancouver, BC V6H 3Z6, Canada; Vancouver Prostate Centre, Department of Urologic Sciences, University of British Columbia, 2660 Oak St., Vancouver, BC V6H 3Z6, Canada; Vancouver Prostate Centre, Department of Urologic Sciences, University of British Columbia, 2660 Oak St., Vancouver, BC V6H 3Z6, Canada; Vancouver Prostate Centre, Department of Urologic Sciences, University of British Columbia, 2660 Oak St., Vancouver, BC V6H 3Z6, Canada; Vancouver Prostate Centre, Department of Urologic Sciences, University of British Columbia, 2660 Oak St., Vancouver, BC V6H 3Z6, Canada; Vancouver Prostate Centre, Department of Urologic Sciences, University of British Columbia, 2660 Oak St., Vancouver, BC V6H 3Z6, Canada; Vancouver Prostate Centre, Department of Urologic Sciences, University of British Columbia, 2660 Oak St., Vancouver, BC V6H 3Z6, Canada; Vancouver Prostate Centre, Department of Urologic Sciences, University of British Columbia, 2660 Oak St., Vancouver, BC V6H 3Z6, Canada; Vancouver Prostate Centre, Department of Urologic Sciences, University of British Columbia, 2660 Oak St., Vancouver, BC V6H 3Z6, Canada; Vancouver Prostate Centre, Department of Urologic Sciences, University of British Columbia, 2660 Oak St., Vancouver, BC V6H 3Z6, Canada; Vancouver Prostate Centre, Department of Urologic Sciences, University of British Columbia, 2660 Oak St., Vancouver, BC V6H 3Z6, Canada; Vancouver Prostate Centre, Department of Urologic Sciences, University of British Columbia, 2660 Oak St., Vancouver, BC V6H 3Z6, Canada; School of Medicine, Koç University, Rumelifeneri Yolu, Istanbul 34450, Turkey; Koç University Research Centre for Translational Medicine (KUTTAM), Koç University, Rumelifeneri Yolu, Istanbul 34450, Turkey; Michael Smith Laboratories, Department of Biochemistry and Molecular Biology, University of British Columbia, Vancouver, BC V6T 1Z4, Canada; Vancouver Prostate Centre, Department of Urologic Sciences, University of British Columbia, 2660 Oak St., Vancouver, BC V6H 3Z6, Canada; Vancouver Prostate Centre, Department of Urologic Sciences, University of British Columbia, 2660 Oak St., Vancouver, BC V6H 3Z6, Canada; Vancouver Prostate Centre, Department of Urologic Sciences, University of British Columbia, 2660 Oak St., Vancouver, BC V6H 3Z6, Canada; Vancouver Prostate Centre, Department of Urologic Sciences, University of British Columbia, 2660 Oak St., Vancouver, BC V6H 3Z6, Canada; Vancouver Prostate Centre, Department of Urologic Sciences, University of British Columbia, 2660 Oak St., Vancouver, BC V6H 3Z6, Canada; School of Medicine, Koç University, Rumelifeneri Yolu, Istanbul 34450, Turkey; Koç University Research Centre for Translational Medicine (KUTTAM), Koç University, Rumelifeneri Yolu, Istanbul 34450, Turkey; Vancouver Prostate Centre, Department of Urologic Sciences, University of British Columbia, 2660 Oak St., Vancouver, BC V6H 3Z6, Canada; Vancouver Prostate Centre, Department of Urologic Sciences, University of British Columbia, 2660 Oak St., Vancouver, BC V6H 3Z6, Canada; Michael Smith Laboratories, Department of Biochemistry and Molecular Biology, University of British Columbia, Vancouver, BC V6T 1Z4, Canada; Vancouver Prostate Centre, Department of Urologic Sciences, University of British Columbia, 2660 Oak St., Vancouver, BC V6H 3Z6, Canada

## Abstract

Numerous cancers, including prostate cancer (PCa), are addicted to transcription programs driven by specific genomic regions known as super-enhancers (SEs). The robust transcription of genes at such SEs is enabled by the formation of phase-separated condensates by transcription factors and coactivators with intrinsically disordered regions. The androgen receptor (AR), the main oncogenic driver in PCa, contains large disordered regions and is co-recruited with the transcriptional coactivator mediator complex subunit 1 (MED1) to SEs in androgen-dependent PCa cells, thereby promoting oncogenic transcriptional programs. In this work, we reveal that full-length AR forms foci with liquid-like properties in different PCa models. We demonstrate that foci formation correlates with AR transcriptional activity, as this activity can be modulated by changing cellular foci content chemically or by silencing MED1. AR ability to phase separate was also validated *in vitro* by using recombinant full-length AR protein. We also demonstrate that AR antagonists, which suppress transcriptional activity by targeting key regions for homotypic or heterotypic interactions of this receptor, hinder foci formation in PCa cells and phase separation *in vitro*. Our results suggest that enhanced compartmentalization of AR and coactivators may play an important role in the activation of oncogenic transcription programs in androgen-dependent PCa.

## INTRODUCTION

Prostate cancer (PCa) is one of the most common cancers in men. Androgen deprivation therapy (ADT) remains the mainstay of systemic therapy for advanced disease. However, progression to castration-resistant PCa (CRPC) over time is inevitable (1,2). A major oncogenic driver of PCa progression to CRPC is the androgen receptor (AR), a ligand-activated transcription factor (3). The AR contains an N-terminus transactivation domain (NTD) that is mainly disordered, a DNA binding domain (DBD), and a C-terminal ligand binding domain (LBD) where androgens such as testosterone or dihydrotestosterone (DHT) bind. Upon androgen binding, AR translocates from the cytoplasm to the nucleus and activates the transcription of target genes. As most PCa patients remain dependent on AR pathway, this transcription factor is the prime target for PCa treatment.

Interactions with transcriptional coactivators, such as MED1, are required for AR activity and increased interactions with these coactivators are associated with metastatic and castrate resistant PCa (4–6). Most recently, it has been revealed that the coactivator MED1 engages AR in androgen-dependent PCa cells at super-enhancer (SE) sites (7), which are clusters of enhancers enriched with RNA polymerase II (RNA POLII), p300 and H3K27ac to cooperatively assemble the transcriptional apparatus at high density and induce robust expression of key genes involved in cell identity (8,9). The interactions between AR and MED1 at SEs and the activation of AR oncogenic transcription programs in PCa cells are both dependent on CDK7-mediated phosphorylation of MED1. Inhibition of MED1 phosphorylation reduces AR-dependent tumor growth (7). These recent findings are in line with multiple studies from the last decade that have identified abnormal SE-driven transcription programs in cancers (10–13).

MED1 has been shown to form nuclear foci at SEs with properties of liquid-like condensates (14). Moreover, an intrinsically disordered region (IDR) in MED1 can form phase-separated droplets *in vitro* that concentrate the transcription apparatus from nuclear extracts (14–16). Based on these observations, it has been suggested that condensates formed by MED1 and other coactivators at SEs may ensure robust transcription of cell-identity defining genes in normal as well as cancer cells (14–16). Within this context, recent discoveries regarding phase separation of AR are of high interest. It has been demonstrated that the intrinsically disordered N-terminal domain of AR forms liquid-like droplets *in vitro* at ∼100 μM (17), and the isolated Tau 5 region within this NTD at ∼500 μM (18). Moreover, the isolated DBD was shown to undergo phase separation in presence of RNA (poly U) (19). Given these recent findings, we hypothesized that AR, both a critical differentiation and oncogenic transcription factor, has the ability to form biomolecular condensates at SEs in androgen-dependent PCa cells as a mean of transcriptional activation.

Using a variety of molecular biology and biophysical approaches (Table 1) we demonstrate that full-length AR forms foci with characteristics of liquid-like condensates in prostate cancer cells upon androgen stimulation. Consistent with this result, full-length AR can phase separate *in vitro*, and this process is inhibited by AR antagonists that target different AR domains. MED1, whose IDR co-localizes with AR condensates *in vitro*, is also essential for AR foci formation in PCa cells. Most importantly, we reveal that AR transcriptional activity in androgen-dependent PCa cells correlates with foci formation, because the AR transcriptional activity follows changes in cellular foci content induced chemically or by cofactor silencing.

## MATERIALS AND METHODS

**Table 1.** Key resources

Reagent or resource	Source	IDENTIFIER
**Antibodies**		
Mouse monoclonal anti-AR	Santa Cruz Biotechnology	Cat#sc-7305
Goat polyclonal anti-AR	MyBioSource	Cat#MBS421183
Rabbit polyclonal anti-MED1	Cell Signaling Technology	Cat#51613
Rabbit polyclonal anti-Phosph-MED1	Abcam	Cat#ab60950
Rabbit polyclonal anti-phospho-RNA Pol II	Abcam	Cat#ab5408
Mouse monoclonal anti-phospho-RNA Pol II	Invitrogen	Cat#MA1-46093
Mouse monoclonal anti-vinculin	Sigma-Aldrich	Cat#V9131
Rabbit polyclonal anti-GFP	Cell Signaling Technology	Cat#2956
Donkey anti-mouse IgG (IRDye 680CW)	LI-COR Biosciences	Cat#926–68072
Donkey anti-mouse IgG (IRDye 800CW)	LI-COR Biosciences	Cat#926–32212
Donkey anti-rabbit Alexa Fluor 594	Invitrogen Life Technologies	Cat#A-21207
Donkey anti-mouse Alexa Fluor 488	Invitrogen Life Technologies	Cat#A-21202
Rat monoclonal anti-BrdU	Abcam	Cat# ab6326
**Bacterial and virus strains**		
One Shot™ TOP10 Chemically Competent E. coli	Invitrogen	Cat#C404003
One Shot™ BL21(DE3) Chemically Competent E. coli	Invitrogen	Cat#C600003
**Biological samples**		
Patient-derived xenografts (PDX) tissue section	Living Tumour Laboratory at Vancouver Prostate Centre	N/A
Tissue Microarray (TMA)	Molecular Pathology core at the Vancouver Prostate Centre	N/A
**Chemicals, peptides, and recombinant proteins**		
Dihydrotesterone	Steraloids	Cat#A2570-000
β-etradiol	Sigma Aldrich	E8875
Progesterone	Sigma Aldrich	P0130
Hydrocortisone	Sigma Aldrich	NIST921A
Enzalutamide	Haoyuan Chemexpress Co	HY-70002
EPI-001	Sigma Aldrich	Cat#SML1844
Tamoxifen	Sigma Aldrich	Cat#T5648
14449	Dr. Cherkasov, VPC	
Bicalutamide	Sigma Aldrich	Cat#1071202
1,6-Hexanediol	Sigma Aldrich	Cat#240117
5-Bromouridine 5′-triphosphate	Sigma Aldrich	Cat#B7166
Actinomycin D	Sigma Aldrich	Cat#A1410
**Critical commercial assays**		
Duolink^®^ In Situ Red Starter Kit Mouse/Rabbit	Sigma-Aldrich	Cat#DUO92101-1KT
CelLytic NuCLEAR Extraction Kit	Sigma-Aldrich	NXTRACT-1KT
QuikChange II Site-Directed Mutagenesis Kit	Agilent Technologies	Cat#200521
**Experimental models: cell lines**		
LNCaP	ATCC	CRL-1740
LAPC-4	ATCC	CRL-13009
293F	Life Technologies	510029
**Oligonucleotides and probes**		
siMED1	Applied Biological Materials	Cat# i5129621
the scramble control siRNA (5′-CAGCGCUGACAACAGUUUCAU-3′)	Dharmacon	N/A-customer designed
*KLK3*: forward (qPCR) 5′- GGGTGTCTGTGTTATTTGTGG-3′	Integrated DNA Technologies	N/A-customer designed
*KLK3*: reverse (qPCR) 5′- TTGCTGTGAGTGTCTGGTG-3′	Integrated DNA Technologies	N/A-customer designed
*FKBP52*: forward (qPCR) 5′- CACTACACTGGCTGGCTATT-3′	Integrated DNA Technologies	N/A-customer designed
*FKBP52*: reverse (qPCR) 5′- TTCACCGCGAGTCTGTATTC-3′	Integrated DNA Technologies	N/A-customer designed
*Vinculin*: forward (qPCR) 5′- TCCTGTAATTCCTACCTCCCTG-3′	Integrated DNA Technologies	N/A-customer designed
*Vinculin*: reverse (qPCR) 5′- TGCCCTCTCATTTTGCCTAG-3′	Integrated DNA Technologies	N/A-customer designed
*TMPRSS2:* forward (qPCR) 5′-GATCTTCCTGCTGAGTCCTTT-3′	Integrated DNA Technologies	N/A-customer designed
*TMPRSS2:* reverse (qPCR) 5′-GAAGGGCTGGTCCCTTTATTT-3′	Integrated DNA Technologies	N/A-customer designed
*KLK3_ChIP_Forward:* 5′-ACAGACCTACTCTGGAGGAAC-3′	ThermoFisher	N/A-customer designed
*KLK3_ChIP_Reverse:* 5′-AAGACAGCAACACCTTTTT-3′	ThermoFisher	N/A-customer designed
*NC_ChIP_Forward:* 5′-CATTTCCTGCTTGTCCTCTG-3′	ThermoFisher	N/A-customer designed
*NC_ChIP_Reverse:* 5′-GGCCTTCCTGGTATGAAATG-3′	ThermoFisher	N/A-customer designed
*TMPRSSS2_ChIP_Forward:* 5′-TTATAAGGCTCAGCCCCAAG-3′	ThermoFisher	N/A-customer designed
*TMPRSSS2_ChIP_Reverse:* 5′-TCAGTGTGTCCATCTTGCAC-3′	ThermoFisher	N/A-customer designed
ROX-ARE-PSA-Forward: 5′- 56-ROXN-GGTCAGCAGGCATCTCTGTTGCACAGATAGAGTGCACAGGTCTGGAGAACAAGGAGTGGGGGGTTATTGGAATTCCACATTGTTTGCTGCACGT -3′	Integrated DNA Technologies	N/A-customer designed
Unlabeled-ARE-PSA-Forward (competitor): 5′- GGTCAGCAGGCATCTCTGTTGCACAGATAGAGTGCACAGGTCTGGAGAACAAGGAGTGGGGGGTTATTGGAATTCCACATTGTTTGCTGCACGT -3′	Integrated DNA Technologies	N/A-customer designed
ARE-PSA-Reverse: 5′- ACGTGCAGCAAACAATGTGGAATTCCAATAACCCCCCACTCCTTGTTCTCCAGACCTGTGCACTCTATCTGTGCAACAGAGATGCCTGCTGACC -3′	Integrated DNA Technologies	N/A-customer designed
ARE-mutant-Forward: 5′- GGTCAGCAGGCATCTCTGTTGCACAGATAGAGTGCACAGGTGTGGGGGGTTATTGGAATTCCACATTGTTTGCTGCACGT-3′	ThermoFisher	N/A-customer designed
ARE-mutant-Reverse: 5′- ACGTGCAGCAAACAATGTGGAATTCCAATAACCCCCCACTACCTGTGCACTCTATCTGTGCAACAGAGATGCCTGCTGACC-3′	ThermoFisher	N/A-customer designed
FKBP5 probe	Empire genomics	WI2-997M11
**Recombinant DNA**		
mEGFP-N1	Addgene	Cat#54767
AR-FL-mEGFP	This paper	N/A
AR-NTD-mEGFP	This paper	N/A
AR-DBD-LBD- mEGFP	This paper	N/A
AR-HLBD-mEGFP	This paper	N/A
AR-V7-mEGFP	This paper	N/A
His-AR-mEGFP	This paper	N/A
AR-mEGFP-MBP-His	This paper	N/A
MED1 cDNA ORF Clone, Human, C-OFPSpark® tag	Sino Biological	HG13221-ACR
Pet28a-MED1-IDR-OFPSpark	This paper	N/A
**Software and algorithms**		
FV31S-SW	Olympus	N/A
ZEN2010	Zeiss	N/A
PRISM	GraphPad	N/A
**Other**		
Lipofectamin 3000	Invitrogen Life Technologies	Cat#L3000015
TransIT2020	Mirus	Cat# MIR4500
Oligofectamin	Invitrogen Life Technologies	Cat#12252011
Antifade mounting medium with DAPI	VECTOR	Cat#H-1200–10
Lipofectin	Invitrogen Life Technologies	Cat#18292037
FastStart Universal SYBR Green Master	Roche	49138500001
Polyethylenimine (PEI 25K)	Polysciences	23966–100
Freestyle 293 expression medium	Gibco	R790-07

### Cell lines

LNCaP and LAPC4 cells were received from the American Type Culture Collection (ATCC). LNCaP and LAPC4 cells were maintained in RPMI-1640 media containing 10% fetal bovine serum (FBS; Invitrogen Life Technologies). All the cell lines were routinely tested for mycoplasma and authentication were confirmed by genomic sequencing or STR profiling.

### Constructs

Non-dimerizing mEGFP-N1 (containing A206K mutation) was a gift from Michael Davidson (Addgene plasmid #54767, RRID:Addgene_54767). AR constructs were cloned at the N-terminus of the mEGFP tag by using the restriction enzyme sites NheI and BamHI. AR mutants were made by QuikChange II Site-Directed Mutagenesis Kit (Agilent Technologies). Med1-IDR (residues 948–1574) was cloned in pET28a with N-terminal OFPSpark tag. For purification purposes, a Histidine tag was added at the N-terminus of AR-mEGFP construct and an AR-mEGFP-MBP-His was cloned in pSF-CMV-PURO-COOH plasmid (Sigma Aldrich).

### Transfection

Transfection of plasmids and siRNAs was carried out using Lipofectamin 3000 and Oligofectamin (Invitrogen Life Technologies), respectively, following the user guide. The siRNAs targeting MED1 were purchased from Applied Biological Materials (Abm, Cat# i5129621) and the scramble was from Dharmacon.

### Immunofluorescence staining and confocal microscopy

Cells grown on glass coverslips were fixed with 3.5% paraformaldehyde (PFA) and then permeabilized with 0.5% Triton X-100. After blocking with 3% skim milk, cells were incubated with the primary antibody diluted in 3% milk (1:200) at 4°C for overnight followed with 1 h incubation of the secondary antibody (1:500) at 37°C for 40 min in the dark. Coverslips were then mounted on slides with DAPI-containing mounting medium (VECTOR). Fluorescence staining was visualized with an Olympus FV3000RS confocal microscope (Olympus Canada Inc., Richmond Hill, Ontario, Canada; manufactured by Olympus in Tokyo, Japan) using a 60x UPLAPO oil objective. The images were taken with the FV31S-SW software with the confocal pinhole set to ‘automatic’ under the Z-stack model. For presentation purposes, images were exported as bitmap (BMP) files. The graph was prepared with the GraphPad Prism 9 software.

### Analysis of the numbers of foci (imaging and quantification)

To quantify the numbers of foci, an imaging map of the whole coverslip was first created using the 4× UPLXAPO lens of the Olympus FV3000RS confocal microscope (Olympus Canada Inc., Richmond Hill, Ontario, Canada; manufactured by Olympus in Tokyo, Japan), and then at least 10 fields were randomly selected from the map for each coverslip for the next step high resolution imaging. The high resolution imaging was taken using the 60x UPLAPO oil lens with the confocal aperture airy disk set to 0.6, averaging to 3, actual scan size as 0.067 μm/pixel, and the z-section ranged to 15 μm and step size optimized to 0.21 μm. The laser power and voltage were set to the same conditions within each experiment. The image processing and quantification were performed using the cellSens software (Olympus) with a customized in-house Marco script. The maximum intensity z-projection was applied to the green channel, and Adaptive Threshold was employed to segment the images for foci identification and quantification. The filter area was set to zero to include all the foci. All experiments were performed with three biological replicates (*N* = 3) and in total 45 cells were quantified. The data is presented as aligned points. For *in vitro* droplets, images were analyzed by cellSens to determine the number of liquid droplet formation, droplet size, number and co-localization using cellSens software. The fluorescence images within a set of experiments were acquired using identical microscope settings (i.e. scan speed, resolution, magnification, optical zoom, gain, offset and laser intensity) to ensure consistency across samples and experimental runs. Thresholding was set to a range of 550–60 000 for green channel, 300–60 000 for red channel and 100–60 000 for gray levels, which effectively eliminate the background signal from the analysis. The pixels within these threshold limits were evaluated for total area (μm^2^), co-localization coefficient and total number of objects in images.

### Analysis of the percentage of cells containing foci (imaging and quantification)

For the quantification of the percentage of cells containing foci, an imaging map of the whole coverslip was created as described above and the high resolution images of at least 10 fields were taken using the 60x UPLAPO oil lens with the confocal aperture airy disk set to 1.0, averaging to 3, actual scan size as 0.276 μm/pixel, and the z section ranged to 15 μm and step size optimized to 0.33 μm. The laser power and voltage were set to the same conditions within each experiment. The image processing and quantification were performed using the cellSens software with a customized in-house Marco script. As described above, the foci were identified and quantified with the Adaptive Threshold segmentation. The nucleus were defined using manual threshold and segmented as Region of Interest (ROI). The numbers of foci for each ROI (nucleus) were count and measured. The ROIs containing at least 20 foci were regarded as foci-positive. All experiments were performed with three biological replicates and a total of 45 fields were analysed for each sample. The data are presented as aligned points.

### Fluorescence recovery after photobleaching (FRAP)

FRAP analysis was performed with a Zeiss confocal laser scanning microscope LSM780 using a Plan-Apochromat 63× 1.40 oil immersion objective. The selected condensates were bleached with the argon laser with 100% of laser power, and the fluorescence recovery was monitored with continuous z-stack scanning for 30 seconds. The images of a non-bleached region were also captured simultaneously from the same field for the reference purpose. A total of 21 foci were monitored for the FRAP assay and the half-lives and mobile fractions of AR-mEGFP-rich condensates were analyzed with Zeiss ZEN software (ZEN2010) and graphed using GraphPad Prism 9 software. For FRAP of *in vitro* droplets, single pulse of laser at 16 ms dwell time was applied to the AR-mEGFP protein in the 488 nm channel. Recovery was imaged on the Zeiss 780 confocal/Olympus FVS3000 microscope using 60x oil immersion objective lens. When recording fluorescence recovery, 512 × 512-pixel images were collected for 50 frames. Fluorescence intensity was measured using Zeiss ZEN software (ZEN2010).

### Proximity ligation assay (PLA)

For the PLA analysis, the protein-protein interaction was identified using a commercially available PLA kit (Duolink; Sigma-Aldrich) as described before (20). The signal was inspected using the Olympus FV3000RS confocal microscope with a 60x UPLAPO oil objective. PLA images were taken using the z-stack model and the PLA signal was quantified with the cellSens software (Olympus). To assess AR and p-MED1 protein-protein interaction in clinical prostate tumor specimens, the Duolink® In Situ Detection Reagents Brightfield Kit (Sigma- Sigma-Aldrich, Oakville, Ontario) was adapted from the manufacturer's instructions for use on the Ventana DISCOVERY Ultra autostainer. Antigen was retrieved at 91°C for 64 min in CC1. Tissues were incubated for 12 h at room temperature with the following antibody cocktail prepared in 1× TBS: Anti-TRAP220/MED1 (phospho T1457) (ab60950, abcam, 1:300) and Anti-AR/Androgen Receptor Antibody (441) (sc-7305, Santa cruz, 1:50). PLA method was conducted as previously described in Zhang *et al.* (21) with the following adaptations to detect AR/p-MED1 interactions: 1 h ligation step and 2 h amplification step. PLA-stained tissue samples were scanned and then scored digitally by a pathologist with Aperio image scope software (Leica Microsystems).

### BrUTP incorporation assay

BrUTP was transfected into cells with Lipofectin (Invitrogen Life Technologies) following the user guide. The transfection solution containing 10 mM BrUTP was incubated with cells for 30 min at 37°C and then replaced with culture medium. Two h later, cells were fixed with 3.5% PFA and stained with anti-BrdU antibody (ab6326, Abcam).

### Immunofluorescence with DNA FISH

LNCaP cells were hormone starved in phenol red free RPMI medium supplemented with 10% Charcoal Stripped Serum (CSS) for 72 h and plated on precoated coverslips with Polyethyleneimine (PEI). Cells were then transfected with AR-mEGFP using TransIT2020 according to manufacture's protocol. Cells were treated, 48 h post-transfection, with either 1 nM DHT or ETOH for 2 h. Cells were then fixed by 4% paraformaldehyde in PBS for 8 min at room temperature. We then washed the cells with PBS three times, permeabilized them with 0.5% Triton X100 for 10 min, and washed again three times with PBS. Cells were then blocked with 4% Bovine Serum Albumin in PBS for 1 h at 37°C and mouse monoclonal anti-AR antibody was added at a concentration of 1:200 for 16 h at 4°C. Cells were then washed with PBS three times and incubated with Alexa-Flour 488 anti-mouse secondary antibody at 1:1000 for 1 h at 37°C. Cells were washed three times with PBS and were then fixed with 4% paraformaldehyde in PBS for 8 min at room temperature. Cells were dehydrated through successive incubations for 2 min each with 70%, 85% and 100% ETOH. Probe mixture was made mixing 2 μl of FKBP5 probe and 8 μl of probe buffer. Five μl of the mixture was placed on glass slides and the coverslips were placed on top where the cell side is toward the probe mixture. Coverslips were sealed using rubber cement. The genomic DNA were then denatured by placing the slides on a hot plate at 75°C for 8 min in the dark. The slides were then incubated at 37°C for 16 h. The coverslips were then removed and washed with 0.3% Igepal/0.4× SSC (Saline Sodium Citrate) for 2 min followed by a final wash with 0.1% Igepal/2× SSC for 2 min. The coverslips were mounted on slides with DAPI-containing mounting medium.

### Quantitative reverse transcription PCR

RNA extraction and reverse transcription PCR (RT-PCR) were performed as described previously (22). Target gene expression was normalized to the levels of Vinculin in respective samples as an internal control.

### Western blot analysis

Proteins (20 μg/well) were separated on SDS-PAGE gels and then transferred to a nitrocellulose membrane (BIO-RAD). Membranes were incubated with a primary antibody (1/1000) at 4°C overnight followed by incubation with a fluorescently labelled secondary antibody (1/5000). Proteins were visualized using the Odyssey Imaging System (LI-COR Biosciences). Vinculin was a loading control. Nuclear concentration of AR was estimated by using a standard curve obtained with recombinant AR protein. Increasing amount of recombinant AR protein were run on SDS Page in parallel with the nuclear extract from AR-mEGFP expressing LNCaP cells. By plotting the intensity of recombinant protein bands as function of concentration, a standard curve was obtained. The intensity of nuclear AR band was then measured and the concentration was derived from the standard curve.

### Chromatin immunoprecipitation (ChIP)-PCR

LNCaP cells were hormone starved in phenol red free RPMI media supplemented with 5% charcoal Stripped Serum (CSS) for 3 days. Cells were then treated with 1nM DHT or ETOH for 2 h and followed by the treatment with 2.5% 1,6-hexanediol or PBS for 30 min. The cells were immediately washed with PBS, cross-linked with 1% formaldehyde, and harvested. Cells were lysed, nuclei extracted, and DNA was sheared to 300–800 base pair (bp) fragments by Bioruptor (Diagenode). After separating 5% of the sample as ‘input’, the crosslinked chromatin was incubated overnight with AR or MED1 antibody conjugated to protein G beads (Dynabeads; Invitrogen) for immunoprecipitation (IP). Chromatin from both IP and ‘Input’ samples were eluted and de-crosslinked at 65°C and DNA was isolated by PCR and DNA cleanup kit (NEB). Enrichment of AR and MED1 at specified enhancers were quantified by qPCR.

### Patient-derived xenografts (PDX) assay

The patient-derived prostate tumor tissue lines were implanted in male NOD-SCID mice supplemented with testosterone (10 mg/mouse), as previously described (23). When tumors reached 500 mm^3^, the animals were sacrificed and tumors were dissected to prepare for the frozen OCT blocks. 5 μm thick tissues were sectioned from the OCT block for the IF staining. The tissues were fixed with ice-cold acetone for 20 min at –30°C and dried at room temperature for 5 min followed with two times washing with Tris-buffered saline (TBS) containing 0.025% Triton X-100 (TBST). The sections were then incubated with background suppressor (23012A, Biotium) for 10 min and 10% normal goat serum (ab7481, Abcam) for 2 h to block the non-specific staining at room temperature. The primary anti-AR antibody was diluted in 1% bovine serum albumin (BSA) in TBST and incubated with the tissues at 4°C for overnight, and the secondary antibody was incubated at 37°C for 1 h in dark. The tissues were counterstained with DAPI (5 μg/ml) in dark for 10 min, washed with TBST for three times and then mounted with ProLong Glass Antifade Mountant (P36982, Invitrogen). Images were taken with a confocal microscope and the percentage of AR foci-containing cells were quantified from 15 fields of each arm from three tumor lines using the method as described above.

### Expression and purification

Full length AR with C-terminal mEGFP and an N-terminal 8xHis tag was cloned in mEGFP-N1 plasmid and AR-mEGFP-MBP-His was cloned in PSF plasmid. Proteins were expressed in FreeStyle 293F cells (Gibco # R790-07), maintained at 37°C and 90 rpm shaking with 8% CO_2_. For the expression 1 μg/μl DNA and 3 μg/μl of Polyethylenimine (PEI 25K) was used to transfect the FreeStyle 293F cells at a density of 1 × 10^6^ cells/ml and >95% viability. After 24 h of transfection 10 μM DHT was added to the transfection media and the cells were harvested 48 h post-transfection. Harvested cells were stored at −80°C until purification.

Cell pellets were thawed on ice and resuspended in buffer A (50 mM Tris, pH 8.0; 150 mM NaCl; 5% glycerol, 20 μM ZnSO_4_; 0.05% NP40; 0.2 mM TCEP; 0.1 mM PMSF and 20 μM DHT) supplemented with 0.1 mg/ml DNaseA, 5 mM MgCl_2_, cOmplete protease inhibitors (Roche, 11873580001) and 1 mM PMSF. After sonication and centrifugation, His-AR-mEGFP and AR-mEGFP-MBP-His were purified by affinity chromatography using Ni-NTA agarose resin and amylose resin, respectively. All the fractions were subjected to gel analysis using 10% SDS-PAGE and confirmed the protein of interest by western blot and mass spectrometry. Fractions containing protein of interest were pooled concentrated (using Amicon Ultra centrifugal filters, 100K MWCO), and injected on size exclusion chromatography (Superose 6) and pure non-aggregated fractions pooled, concentrated and used to study the *in vitro* droplet formation.

### *In vitro* droplet assay

Recombinant His-AR-mEGFP and AR-mEGFP-MBP-His proteins were concentrated to an appropriate protein concentration. Recombinant proteins in assay buffer (50 mM Tris, pH 8.0; 150 mM NaCl; 5% glycerol; 20 μM ZnSO_4_; 0.05% NP40; 0.2 mM TCEP; 0.1 mM PMSF and 10 μM DHT) was mixed with 10% crowding agent (PEG-8000). Ten μl protein solution was loaded onto clean rectangular cover glass (VWR, # 48404-452) and imaged with Olympus FVS3000 confocal microscope with 60× UPLAPO objective and oil immersion. Images were acquired at 2X zoom and 256 × 256 pixels. 10 frames were collected for each image and five different fields were studied and imaged for each drop. The droplet formation was studied at different time points; different concentrations of protein; in presence of different concentrations of crowding agent, salt and inhibitors. Assay buffer and purified recombinant GFP protein were used as negative control in all the experiments. For AR and MED1 *in vitro* interaction, purified His-AR-mEGFP or AR-mEGFP-MBP-His was incubated with purified MED1-IDR-OFPSpark (residues 948–1574; protocol adapted from Sabari *et al.* (14)), in presence of 10% PEG-8000. Ten μl of the mixture was then added to the coverslip and droplet formation was evaluated under the microscope after 20 min incubation at room temperature. In experiments in which liquid phase separation of AR in presence of androgen response element (ARE) of the PSA promotor was examined, recombinant mEGFP-MBP-His tagged AR protein was mixed with 12.5 nM ROX labeled double stranded DNA and 10% PEG-8000. The reaction was then added on a cover glass and formation of AR droplets was visualized after 20 min. For competition studies, either the unlabelled ARE-PSA (50×) or mutant lacking the PSA ARE (50×) were used.

### Turbidity assay

Turbidity of protein solutions was determined as the absorption at 340 nm using a Nanodrop OneC instrument (Thermo Fisher). Turbidity was measured after 20 min incubation at room temperature. All the readings were taken in presence of 10% PEG-8000 except when studying the effect of different concentrations of PEG.

### AR recruitment to SE sites

The H3K27ac ChIP-seq data from LNCaP cells treated with DHT were downloaded from GEO (GSM3358196) and reanalyzed to call super-enhancers. Briefly, raw FASTQ files were aligned to hg19 genome-build using BWA-MEM (v0.7.1) default options. MAPQ <30 alignments were filtered out, and duplicates were removed according to the ‘samtools markdup’ (v1.16) manual page. Peaks are called using MACS3 (v3.0.0a7) that was controlled with input DNA (GSM1249450). Finally, ‘.narrowPeak’ files were converted to ‘.gff’ format as input for the ROSE program with custom scripts. AR (GSM3567213) and MED1 (GSM3567216) BigWig files were downloaded from ChIP-atlas (http://chip-atlas.org). To compare AR and MED1 occupancy in super- and non-super enhancers, BigWig signal was captured by bluegill (http://bream.bio) and then RPM normalized.

### Statistics

Statistical analyses were performed in GraphPad Prism 9 using one-way ANOVA and two-tailed unpaired Student's *t*-test. *P* values are indicated by stars: ns ≥ 0.05, * 0.01 to 0.05, ** 0.001 to 0.01, *** 0.0001 to 0.001, **** < 0.0001.

## RESULTS

### AR-rich nuclear foci are formed in PCa models upon androgen stimulation

**Figure 1.**
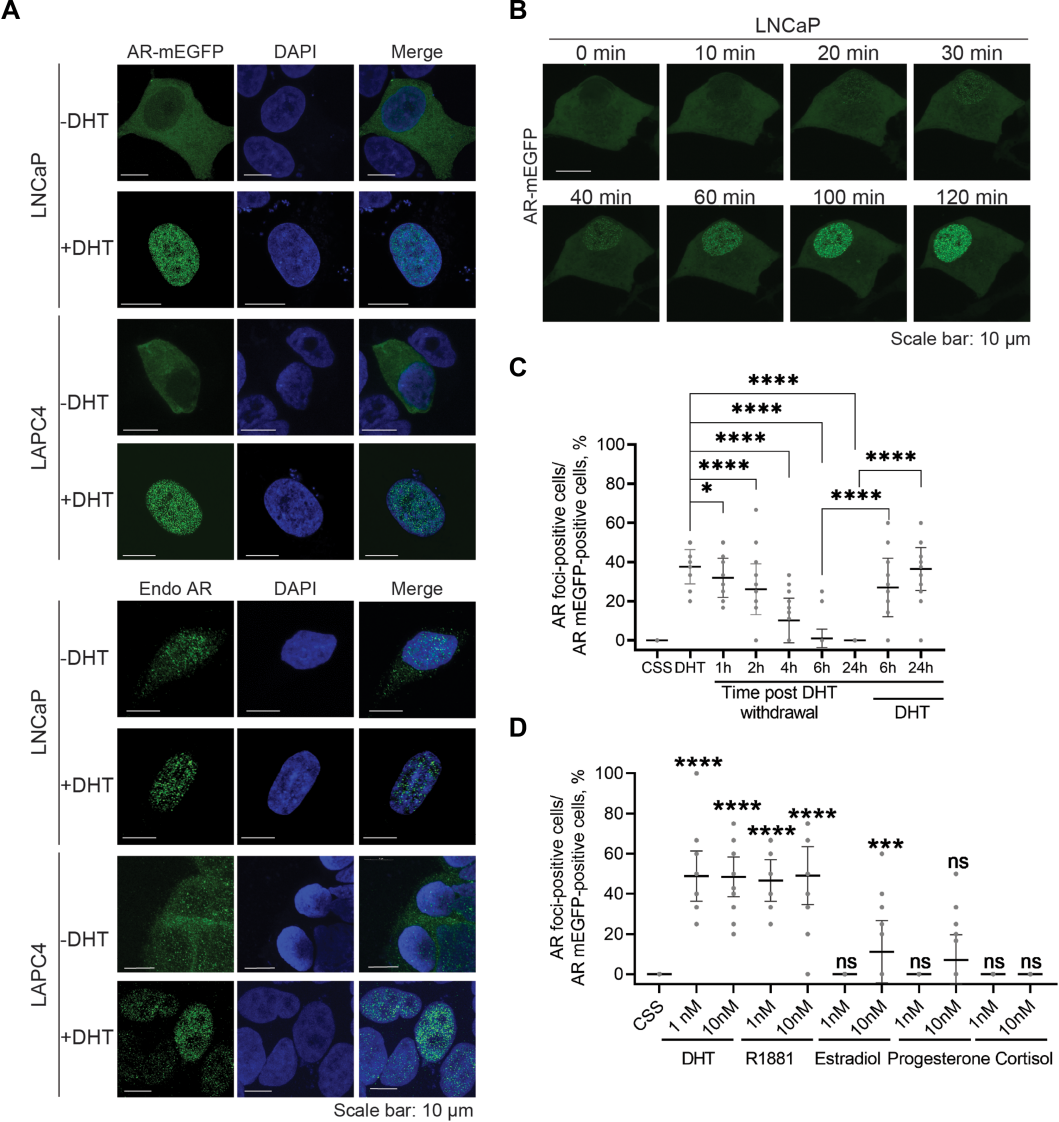
AR foci form upon androgen stimulation. (**A**) Foci formed by transiently expressed and endogenous AR (Endo AR). Cells were cultured with 5% CSS-containing medium for two days then stimulated with 1 nM DHT or ethanol (control) for 2 h. The transiently expressed AR-mEGFP and the immune-stained endogenous AR were inspected under confocal microscope. (**B**) Time-lapsed foci formation. LNCaP cells expressing AR-mEGFP were hormone starved (5% CSS) for 2 days and then stimulated with 1 nM DHT. A live time-lapse confocal imaging assay was performed along the DHT treatment. (**C**) Reversibility of foci formation. AR-mEGFP expressing LNCaP cells were starved in 5% CSS for 2 days, treated with DHT for 2 h, and then washed twice to remove DHT. Cells were then cultured in 5% CSS for the indicated time course. DHT was applied back to cells at 6h and 24 h post-DHT removal, respectively. AR-rich foci were quantified. Percentage of AR-mEGFP foci containing cells were quantified from 45 fields of three independent experiments and the data was presented as aligned points with mean ± SD. (**D**) Androgen dependence of foci formation. AR-mEGFP expressing LNCaP cells were cultured in 5% CSS for 2 days then treated with various hormones and foci formation was quantified as in 1C. One-way ANOVA was used to analyse the data in (C) and (D). *P* values are indicated by stars: ns ≥ 0.05, * 0.01 to 0.05, ** 0.001 to 0.01, *** 0.0001 to 0.001, **** < 0.0001.

A recent report showed that 65% of transcriptionally active SEs in androgen-dependent PCa cells are co-enriched in AR and MED1 upon DHT stimulation (7). Together with previous findings of phase separation capability of MED1 (14), these data suggest that the AR may be recruited to transcriptionally active condensates at SEs in androgen-dependent PCa cells. To investigate this hypothesis, we first probed the ability of full-length AR to form foci upon androgen stimulation in PCa cells. We thus evaluated whether AR tagged with a non-dimerizing Enhanced Green Fluorescent Protein (AR-mEGFP) forms foci in LNCaP and LAPC4 cell lines. Upon androgen stimulation, AR-rich foci were seen in the nucleus of LNCaP and LAPC4 cells (Figure 1A). The empty vector expressing the mEGFP protein itself did not show foci formation in presence or absence of androgens (Supplementary Figure S1A). We also confirmed that endogenous AR forms foci in these PCa cell lines upon DHT stimulation using immunofluorescence (IF) staining with anti-AR antibody (Figure 1A). We could see some foci in the absence of androgens, most probably due to incomplete androgen depletion from the media, however the number of foci increased significantly upon androgen stimulation. Endogenous AR also formed foci like structures in patient derived xenograft (PDX) PCa tissues (Supplementary Figure S1B) that were significantly reduced post castration (Supplementary Figure S1C).

To investigate the reversibility of foci formation, we followed AR-rich foci by a time-lapse live imaging assay in LNCaP cells (Figure 1B and Movie S1). Foci containing AR-mEGFP started to form at 30-min (min) to 1 h post-DHT stimulation, consistent with the time previously reported for AR nuclear translocation (24). The number of cells with foci peaked at about 2 h (Supplementary Figure S1D), and dropped rapidly after DHT removal from the medium, with nearly no foci left after 6 h (Figure 1C). However, if the DHT was added back to the media, the foci formed again (Figure 1C), suggesting that foci formation is sensitive to the environmental androgens and is highly dynamic and reversible. We also investigated if other steroid hormones can stimulate AR foci formation. AR-rich foci were specifically induced by the androgens DHT and R1881 but only very weakly by high concentrations of estradiol or progesterone (Figure 1D). This specificity in foci formation is consistent with the androgen dependence of AR transcriptional activity that we reported previously (25). Taken together, our findings demonstrate that AR foci reversibly form in androgen-dependent PCa cells specifically upon androgen stimulation.

### AR-rich foci exhibit properties of liquid-like condensates

**Figure 2.**
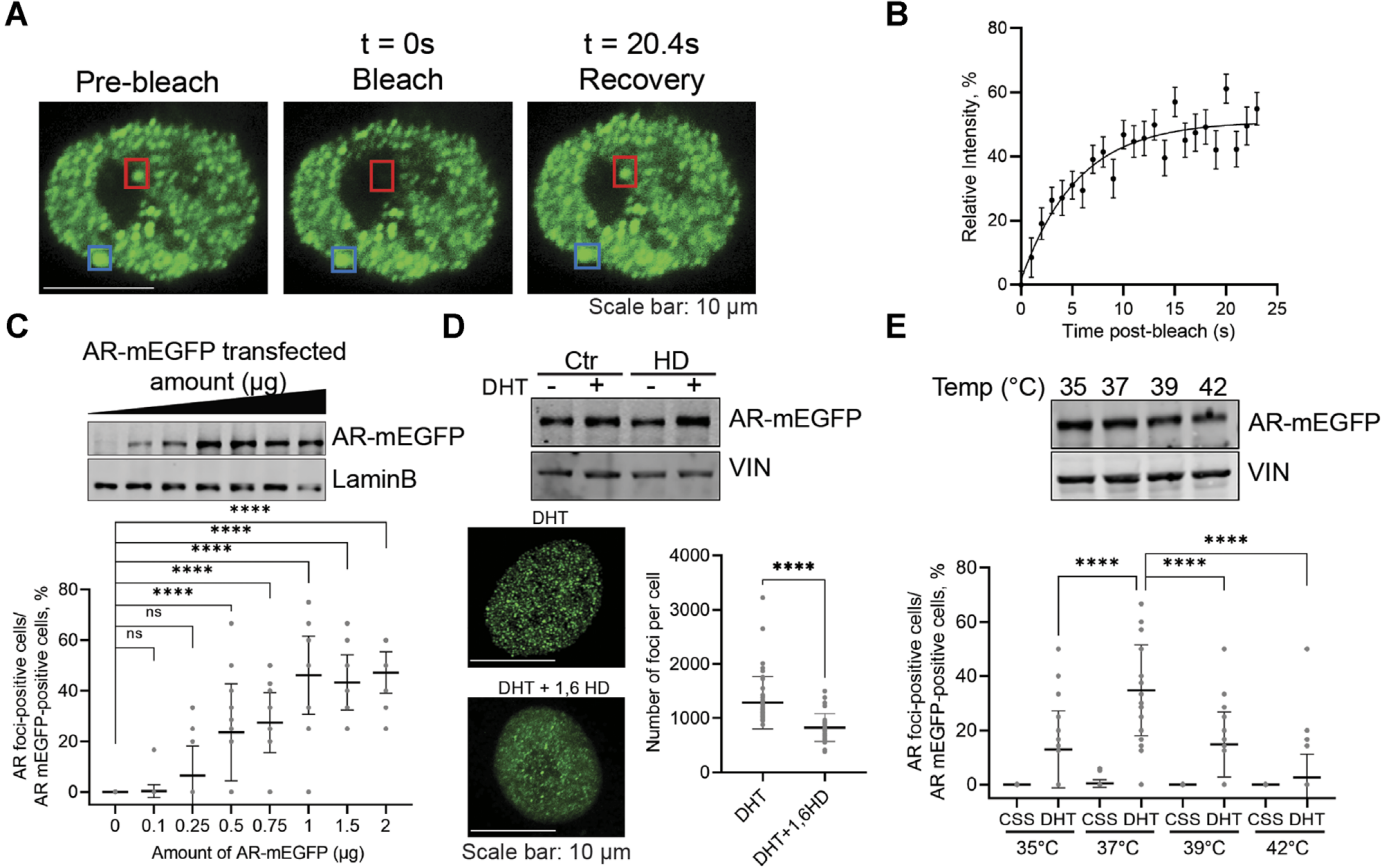
AR foci present liquid-liquid condensate characteristics in AR-mEGFP expressing LNCaP cells. (**A**, **B**) FRAP assay to examine the diffusion of AR in and out the condensates. Cells were cultured in 5% CSS media for 2 days, then stimulated with 1 nM DHT for 2 h. Foci were photobleached with 100% of laser power for 10 s, the time-lapse imaging on the bleached punctum (red) as well as a reference punctum (blue) were captured with confocal microscope in a Z-stacking model (A). FRAP results are presented as mean ± SD (21 cells from 3 biological replicates) (B). (**C**) Foci numbers as a function of AR expression levels. Cells transfected with increasing amount of AR-mEGFP plasmid were cultured in 5% CSS media for 2 days and then stimulated with 1 nM DHT for 2 h. AR-rich foci were quantified by confocal microscopy. Data are presented as aligned points and the horizontal bar corresponds to the mean ± SD (45 fields were analysed from 3 biological replicates). The corresponding nuclear AR levels were evaluated by western blot (WB, anti-GFP antibody). (**D**) AR Foci's response to 1,6-hexanediol (HD). Cells cultured in 5% CSS for 2 days were stimulated with DHT for 2 h, treated with 4% 1,6-HD for 5 min and then fixed. The effect of 1,6-HD on AR protein level was evaluated by WB. The numbers of foci were quantified from 45 cells from three biological replicates and the data is presented as mean ± SD. The graph presents aligned points. (**E**) Foci formation at different temperatures. Cells starved in 5% CSS for 2 days were cultured at various temperatures for 1 h then 1 nM DHT was added for an additional h and AR-rich condensates were quantified. The effect of temperatures on the stability of AR protein was evaluated by WB. The aligned dotted blot presents the data from 45 fields from three independent experiments with mean ± SD. *P* values are indicated by stars: ns ≥ 0.05, * 0.01 to 0.05, ** 0.001 to 0.01, *** 0.0001 to 0.001, **** < 0.0001.

Next, we investigated whether AR foci have characteristics consistent with liquid-like condensates. Hallmarks of such condensates are rapid internal reorganization and exchanges with the surrounding proteins from the dilute phase which can be probed via fluorescence recovery after photobleaching (FRAP) (14,26,27). We observed that AR-mEGFP foci recovered fluorescence after photobleaching on a time scale of seconds with an average half time of *t* = 3.80 s (95% CI: 2.61–5.98 s) and a mobile fraction of 51.01% of the reference signal (*n* = 21 foci from 15 cells; 95% CI: 46.84–57.24) (Figure 2A, B and Supplementary Figure S2A). However, some of the condensates did not fully recover their fluorescence in the measured time frame, thus the overall 49% of calculated immobile fraction. We also evaluated the ability of MED1-mEGFP foci to recover fluorescence after photobleaching in the same LNCaP cell model, and found that MED1-mEGFP foci have an average half time of *t* = 3.08 s (95% CI: 2.61–3.67 s) and a mobile fraction of 96.26% of the reference signal (*n* = 24 foci from 24 cells; 95% CI: 93.12–99.88) (Supplementary Figure S2B).These results are consistent with previous FRAP measurements for coactivator MED1 in embryonic stem cells (14). The percentage of cells presenting AR-rich foci increases quickly with the transfected amount of AR-mEGFP plasmid and plateaued at 1 μg, corresponding to a nuclear AR-mEGFP concentration of 5.5 ± 0.8 μM, estimated using a standard curve with purified AR protein (Figure 2C and Supplementary Figure S2C). This result is reminiscent of phase behaviour previously measured for G3BP1 (28–30). We then tested the effect of 1,6-hexanediol (1,6-HD), a compound known to disrupt liquid-like condensates (14,26), on the formation of AR-rich foci in LNCaP cells. Following HD treatment, the number of foci in cells was significantly reduced but the nuclear AR protein levels remained unchanged (Figure 2D). A key feature of condensates is that the saturation concentration for their formation is temperature dependent (27). To investigate if AR foci are responsive to temperature changes, we stimulated AR-mEGFP transfected LNCaP cells with DHT and incubated them under various temperatures for 1 h, and then fixed them for quantification by confocal microscopy. The temperature was confirmed by using a control sample with the same volume and incubation time as the assay samples. Foci formation was maximal at 37°C and decreased significantly at lower and higher temperatures with no significant effect on AR protein levels (Figure 2E). Overall, these results indicate that AR foci display features consistent with liquid-like condensates.

### Full-length AR forms liquid-like droplets *in vitro*

**Figure 3.**
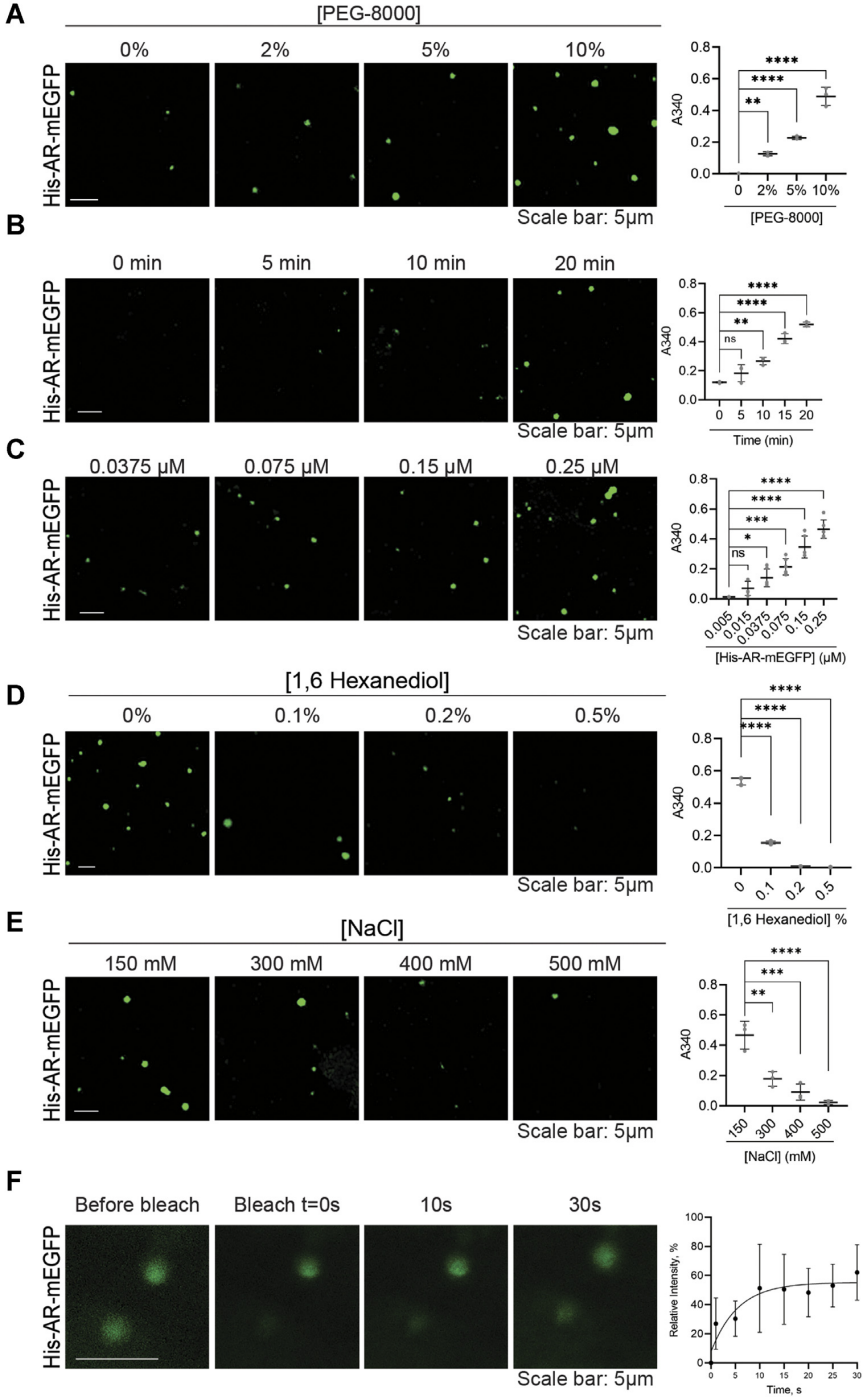
Recombinant AR protein forms droplets *in vitro*. (**A**) Effect of PEG-8000 concentration on His-AR-mEGFP (0.25 μM) droplet formation as characterized by confocal microscopy and turbidity assay after 20 min at room temperature. Droplet formation is associated with increased turbidity and light scattering that could be followed by optical density measurements at 340 nm. (**B**) Droplets as a function of time. Droplets are formed 15–20 min after incubation with 10% PEG-8000. (**C**) Droplets as function of His-AR-mEGFPconcentration. The number of droplets increased with the increasing concentrations of His-AR-mEGFP. Effect of 1,6-hexanediol (**D**) and salt concentration (**E**) on His-AR-mEGFP droplet formation. His-AR-mEGFP at 0.25 μM was incubated for 20 min in presence of 10% PEG-8000 and increasing concentration of 1,6-hexanediol or NaCl. (**F**) FRAP assay showing fluorescence recovery after photobleaching of His-AR-mEGFP droplet in comparison to an unbleached droplet and the relative fluorescence intensity of the bleached droplets (mean ± SD, *N* = 3). Scale bars: 5 μm. *P* values are indicated by stars: ns ≥ 0.05, * 0.01 to 0.05, ** 0.001 to 0.01, *** 0.0001 to 0.001, **** < 0.0001.

To evaluate the ability of the AR to phase separate *in vitro*, we next expressed and purified DHT activated full length AR-mEGFP tagged with an N-terminal poly-histidine tag or a C-terminal maltose binding protein (MBP)-His tag, the latter allowing for higher purity levels (Supplementary Figure S3A). Both, recombinant His-AR-mEGFP (Figure 3) and AR-mEGFP-MBP (Supplementary Figure S3) formed spherical assemblies in the presence of the crowding agent polyethylene glycol (PEG) 8000 and at similar AR concentrations, as detected by confocal microscopy and by a turbidity assay that measures the optical density at 340 nm, a wavelength that is very sensitive for protein assemblies as it measures mainly light scattering of the molecules far from the absorbance of protein aromatic residues (27,31) (Figures 3 and Supplementary Figure S3). The assemblies showed the expected spherical shape of droplets and their formation was optimal in the presence of 10% PEG-8000 and after 20 min incubation time (Figure 3A, B and Supplementary Figure S3B). The lever rule predicts droplets’ volume fraction to increase with growing concentration of components in the system. Therefore, we performed experiments with increasing concentrations of full-length AR. Droplets showed concentration dependent density distributions (Figure 3C and Supplementary Figure S3C) at concentrations above 0.01 μM. The size and number of droplets significantly increased in the studied concentration range (Figure 3C and Supplementary Figure S3C). By contrast, recombinant GFP protein alone did not show any droplet formation in the presence of 10% PEG-8000 even at concentrations ten times higher than the AR (Supplementary Figure S3D).

To probe the biophysical properties of the droplets, we tested their ability to form under different salt and 1,6-HD concentrations and their diffusive properties by FRAP. Similar to observations made for *in vitro* droplets formed by MED1 (14), glucocorticoid receptor GR (31), or estrogen receptor ER (32), the size and numbers of AR droplets were reduced by increasing 1,6-HD concentrations (Figure 3D and Supplementary Figure S3E). The effect of salt was more pronounced in reducing His-AR-mEGFP droplets compared to AR-mEGFP-MBP, most probably due to the solubilizing characteristic of the MBP tag (Figure 3E and Supplementary Figure S3F). These results suggest that *in vitro* condensate formation by AR is driven by a variety of molecular interaction types. Similar to NUP98 (33), performing FRAP *in vitro* was challenging due to the rapid movement of the droplets in solution, and the absence of recovery of the stagnant ones. Probed His-AR-mEGFP- droplets showed fluorescence recovery after photobleaching on a time scale of seconds with half time of *t* = 4 s, however the mobile fraction was only around 55% suggesting a fast transition to a gel-like behaviour (Figure 3F). Indeed, AR-mEGFP-MBP occasionally forms more amorphous assemblies, particularly at high concentrations. Overall, these results demonstrate that full-length AR is able to form liquid-like condensates by its own *in vitro*.

### Multiple domains are required for foci formation by AR in PCa cells

**Figure 4.**
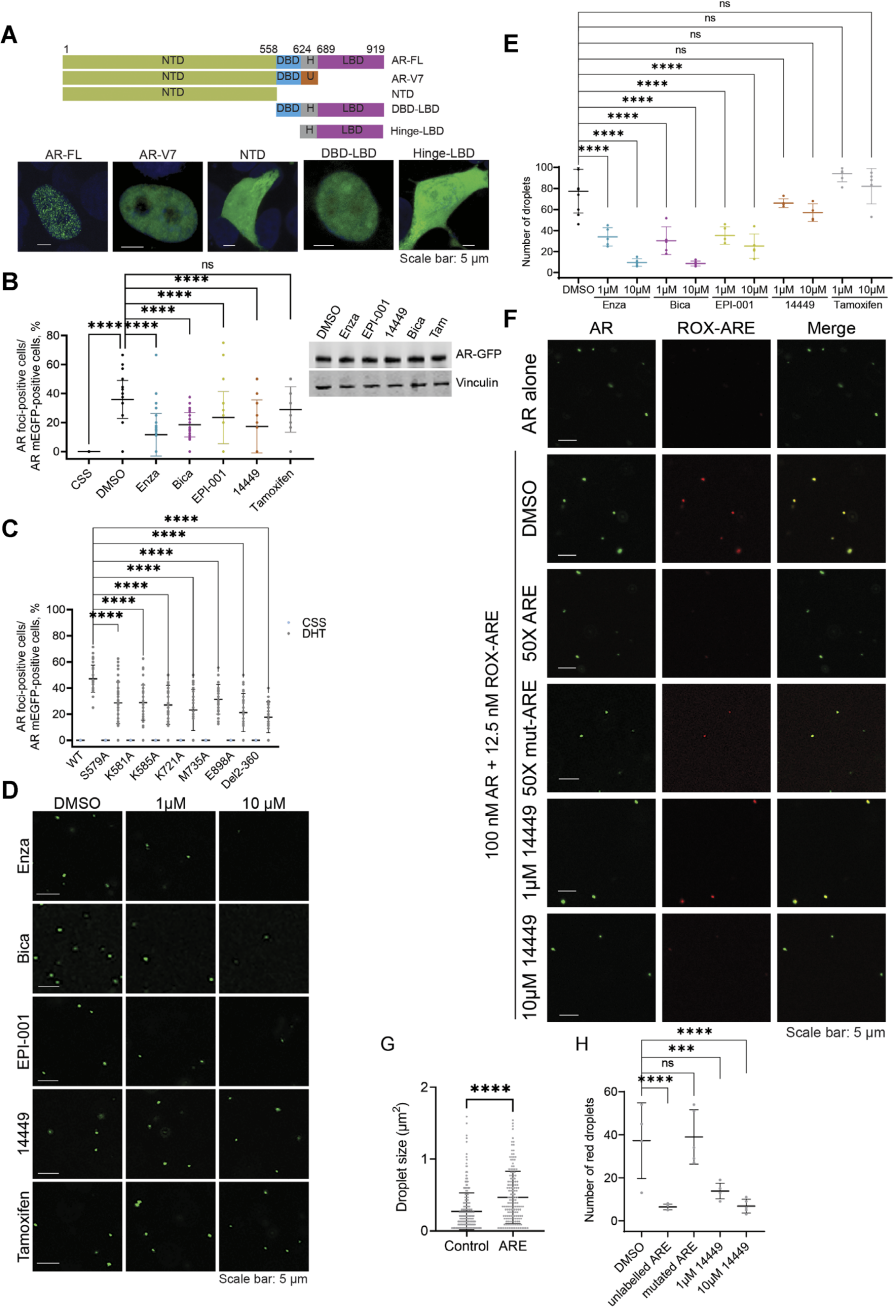
Full Length AR is required for foci formation in LNCaP cells. (**A**) (Top) Different truncated forms of AR-mEGFP: Full length (FL), N-terminal domain (NTD), DNA-binding domain (DBD), hinge region (H) and the ligand-binding domain (LBD). AR variant 7 protein (AR-V7) presents its unique cryptic exon inclusion sequence (U). (Bottom) Foci formation in LNCaP cells transiently transfected by mEGFP tagged truncated AR, starved in 5% CSS for 2 days, then stimulated with 1 nM DHT for 2 h. (**B**) Impact of AR antagonists on foci formation in LNCaP cells. AR-mEGFP transfected LNCaP cells starved in 5% CSS for 2 days were stimulated with DHT for 2 h and then received treatment with 10 μM of enzalutamide (Enza), bcalutamide (Bica), EPI-001, 14449 and Tamoxifen for 2 h. The aligned points present the data from 45 fields and from three independent experiments with mean ± SD. (Right panel) Western blot showing nuclear AR-mEGFP levels 2 h post-treatment with the studied inhibitors. (**C**) Effect of mutations or truncation that either disrupt DNA binding or AR N/C interactions on AR foci formation. LNCaP cells were transfected with mEGFP tagged AR constructs and starved in 5%CSS for 2 days then stimulated with DHT for 2 h. Foci formation was quantified and presented as in B. (**D**) Disruption of AR droplet formation by AR antagonists EPI-001, bicalutamide, 14449 and enzalutamide *in vitro*. Inhibitors were incubated with AR protein (0.1 μM) in presence of 10% PEG-8000 for 20 min prior to visualization by confocal microscopy. Tamoxifen was used as negative control. (**E**) Quantification of the impact of antagonists on the number of AR droplets. The data corresponds to the mean ± SD (*N* = 5). (**F**) Colocalization of AR-mEGFP-MBP (0.1 μM) and ROX labelled ARE (12.5 nM) and the effect of unlabeled or mutated ARE as well as DBD inhibitor (14449) on this colacalization. (**G**) Effect of ARE addition on AR droplets size. (**H**) Quantification of the effect of unlabeled or mutated ARE and 14449 on DNA recruitment to AR droplets. Data are presented as aligned points with mean ± SD (*N* = 5). *P* values are indicated by stars: ns ≥ 0.05, * 0.01 to 0.05, ** 0.001 to 0.01, *** 0.0001 to 0.001, **** < 0.0001.

Biomolecular condensate formation appears to be a common property of intrinsically disordered activation domains of transcription factors and coactivators such as MED1 (14,16). However, recent studies revealed that the essential property of scaffolds that drive the formation of phase separated stress granules is their multi-domain character (34). In order to identify which AR domains are necessary for foci formation in PCa cells, we generated mEGFP-tagged AR truncations and tested foci formation upon DHT stimulation and validated that all proteins were expressed to similar levels (Figure 4A and Supplementary Figure S4A). Foci were only seen in presence of full-length AR after DHT stimulation and not with any of the truncated domains (Figure 4A and Supplementary Figure S4B). Even the constitutively active AR splice variant-7 (AR-V7), which lacks the LBD, did not form foci, suggesting an important role of the LBD in this mechanism. This finding is consistent with a recent work showing only a very mild ability of V7 to form foci (35).

To test the importance of individual domains for foci formation, we used small molecule inhibitors that disrupt specific AR interactions. We tested the effect of AR antagonists that target the NTD (EPI-001) (36), DBD (14449) (37) or the LBD (enzalutamide: Enza and bicalutamide: Bica) on foci formation (38). EPI-001, bicalutamide and enzalutamide inhibit homotypic (N/C-terminal) as well as heterotypic (coactivator) protein interactions by AR (39–41) that may be required for foci formation, respectively, phase separation in PCa cells. The inhibitor 14449 impedes AR’s DNA binding activity, thus enabling a test on the importance of AR recruitment to DNA in foci formation. As a negative control, we used the ER antagonist tamoxifen. AR-mEGFP transfected LNCaP cells were stimulated with DHT for 2 h to ensure AR nuclear translocation and foci formation, and then treated with 10 μM of the tested drugs for an additional 2 h. Foci formation was significantly reduced 2 h post-treatment with enzalutamide, bicalutamide, 14449, and EPI-001 compared to the vehicle control, with no significant effect on the nuclear levels of AR (Figure 4B and Supplementary Figure S4C, D). As expected, tamoxifen had no significant effect on AR foci. Similar effects were seen when LNCaP cells were treated with AR antagonists prior to androgen treatment, except for enzalutamide that inhibited foci formation more significantly (Supplementary Figure S4E). This additional effect could be attributed to reduction of AR nuclear translocation by enzalutamide (42).

Certain AR mutants found in patients convert bicalutamide and enzalutamide into agonists (25,43). Therefore, we also evaluated the effect of these drugs on foci formation of AR mutants W742L and F877LT878A. The mEGFP-tagged mutants as well as the wild type AR plasmids were transfected into Charcoal Stripped Serum (CSS) starved LNCaP cells. As expected, no foci were formed with the wild-type AR in the presence of these drugs. However, enzalutamide and bicalutamide induced F877L/T878A-rich and W742L-rich foci, respectively, in a dose dependent manner (Supplementary Figure S4F), which is concordant with previously reported transcriptional activity associated with those mutants. Homotypic interactions between FXXLF motifs in the NTD and the C-terimnal LBD of the AR (N/C interactions) as well as DNA binding are key to AR’s transcriptional activity. To asses the importance of these interactions for foci formation, we also tested the effect of AR point mutations in the DNA binding pocket (S579A, K581A and K585A) (44), the FXXLF binding pocket in the LBD (K721A, M735A and E898A) (45) as well as the removal of the N-terminal ^23^FXXLF^27^ and ^178^LXXIL^182^ motifs (Del 2–360) on AR foci formation. Interfering with both, DNA binding or N/C interactions, significantly decreased AR's ability to form foci in LNCaP cells (Figure 4C and Supplementary Figure S4G).

As EPI-001, bicalutamide, and enzalutamide inhibit N/C (homotypic) interactions, we also tested the effect of these AR antagonists on droplets formation *in vitro* (Figure 4D, E) using the higher purity AR-mEGFP-MBP construct. All three AR antagonists inhibited droplet formation, indicating that N/C interactions are important for phase separation of recombinant full-lenth AR. Interestingly, 14449 had a minimal effect *in vitro*, similar to the negative control tamoxifen (Figure 4D, E). Taken together, cellular and *in vitro* data suggest that interactions mediated by the LBD and NTD are required for phase separation while the DNA-binding interface of AR may be necessary for the recruitment to androgen response element (ARE) in the genome and the formation of foci at these elements. To evaluate the effect of 14449 further, we tested whether the ARE from the promotor of *KLK3* gene is recruited to AR droplets and whether 14449 can impede this recruitment. As seen in Figure 4F, ROX-labelled ARE from the promotor of the *KLK3* gene indeed colocalized with AR-mEGFP-MBP droplets. Importantly, the addition of ARE significantly increased the size of AR droplets (Figure 4F, G) and unlabelled ARE but not a mutated sequence lacking the ARE site (50x excess) impeded the recruitment of ROX-ARE to AR droplets (Figure 4F and H). The AR antagonist 14449 mirrored the effect of unlabelled DNA and prevented ROX-ARE recruitment to AR droplets as shown by reduction of the red droplets (Figure 4F and H).

### AR foci formation correlates with transcriptional activity in PCa cells

**Figure 5.**
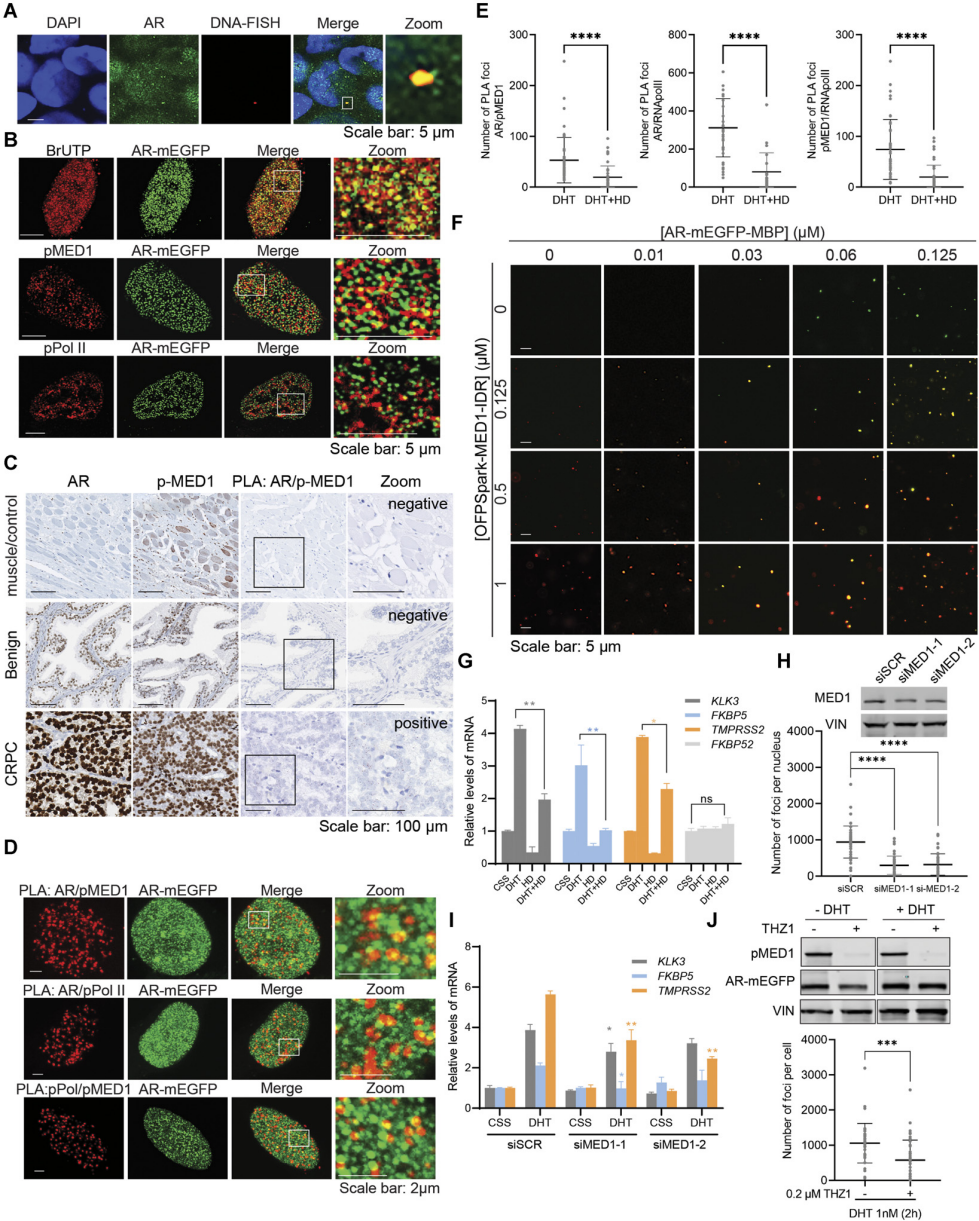
Foci formation correlates with AR transcriptional activity in mEGFP expressing LNCaP cells. (**A**) Co-localization between AR foci and the locus of *FKBP5* as detected by immunofluorescence (IF) and DNA-FISH in LNCaP cells transfected with AR-mEGFP, starved for 2 days then stimulated with 1 nM DHT for 2 h. The merge between DAPI staining (blue), AR foci (green) and *FKBP5*-FISH signal (red) is shown. The area of colocalization is enlarged in the zoom panel. (**B**) IIF imaging of BrUTP, pMED1 and pPol II. LNCaP cells transfected with AR-mEGFP were starved with 5% CSS for two days and then stimulated with 1 nM DHT for 2 h. For BrUTP incorporation assay (top panel), cells were incubated with BrUTP transfection solution for 30 min and then incubated with culture medium without BrUTP for another 2 h before fixation. IF on BrUTP, pMED1 and pPol II were performed (red panels) and the co-localization with AR-rich foci (green panel) were examined under confocal microscope. Images in the white frames were enlarged and displayed in the right panel. (**C**) The expression levels of AR and pMED1 were assessed in clinical prostate tumor specimens with the Ventana DISCOVERY Ultra autostainer. The interaction between the two proteins was evaluated by proximity ligation assay (PLA). (**D**) Colocalization of combined PLA staining (red) and AR-mEGFP (green) in LNCaP cells starved in 5% CSS for 2 days then stimulated with 1 nM DHT. Images in the white frames were enlarged and displayed in the right panels. (**E**) AR-mEGFP transfected LNCaP cells were grown in 5% CSS for two days and then received 1 nM DHT for 2 h. Cells were then treated with or without 4% 1,6-hexanediol (HD) for 5 min before fixing and PLA staining. The quantification of PLA signal was performed using the same method as foci quantification. The aligned points present the data from 45 cells and from three independent experiments with mean ± SD. (**F**) AR-mEGFP-MBP droplet and MED1-IDR colocalization *in vitro*. (**G**) Effect of HD on mRNA levels of different genes. Cells were starved for 3 days and then treated with 1 nM DHT ± 2.5% HD for 30 min, and then washed and incubated with DHT containing medium for 16 h. Total RNAs were extracted and the mRNA levels of genes of interest were examined using qRT-PCR. Values are expressed as mean ± SD. (**H-I**) Effects of knocking down of MED1 on AR condensates (H) and on AR transactivation (I). (**H**) LNCaP cells were transfected with AR-mEGFP plasmid and 6 h later with siRNA targeting MED1 (siMED1) or control (siSCR). Cells were then grown in 5% CSS for 2 days and stimulated with 1 nM DHT for 2 h. Cells were fixed and the foci formation was quantified. The reduction of MED1 protein levels was validated by western blot. Vinculin (VIN) was used as a loading control. The aligned points represent data from 45 cells from three independent experiments. (**I**) Cells transfected with siSCR or siMED1 were grown in 5% CSS media for 3 days and stimulated with 1 nM DHT for 16 h. Levels of AR-targeting genes were examined with q-RT-PCR. The PCR values are expressed as mean ± SD. (**J**) Inhibiting MED1 phosphorylation by THZ1 (0.2 μM for 2 h) reduces foci formation without affecting AR protein levels. LNCaP cells transfected with AR-mEGFP plasmid were cultured in 5% CSS for 2 days and then treated with or without 0.2 μM THZ1 for 2 h before the stimulation with 1 nM DHT for 2 h. MED1 phosphorylation and AR-mEGFP protein levels were investigated with WB. The number of Foci formation per nucleus was quantified and presented with aligned dotplot as mean ± SD from 45 cells and from three independent experiments. p values are indicated by stars: ns ≥ 0.05, * 0.01 to 0.05, ** 0.001 to 0.01, *** 0.0001 to 0.001, **** < 0.0001.

Our results demonstrate that AR can form condensates both *in vitro* and in PCa cells that have liquid-like properties. However, whether such dynamic assembles could have functional relevance is not clear. Therefore, we aimed to determine if foci formation is associated with AR transcriptional activity. Therefore, we first confirmed using a DNA-FISH probe the colocalization of AR-rich foci with *FKBP5*, an AR target gene associated with SE programs (Figure 5A). We then studied the colocalization of AR foci with nascent RNA (Figure 5B, Supplementary Figure S5A). AR-mEGFP transfected LNCaP cells were stimulated with DHT for 2 h, labelled with 5-Bromouridine-5′-triphosphate, fixed and stained. The newly synthesized RNA was visualized using an anti-Bromodeoxyuridine (BrdU) antibody. As seen in Figure 5B (top) and Supplementary Figure S5A, the majority of AR foci colocalized with nascent RNA. Similar colocalization was also found with endogenous AR (Supplementary Figure S5B). Blocking active transcription with actinomycin D (46), reduced AR foci formation (Supplementary Figure S5C) and the mRNA levels of AR target genes (Supplementary Figure S5D).

MED1 interacts with AR and facilitates the recruitment of RNA polymerase II (Pol II) and the initiation of transcription (7,47). Androgen stimulation in PCa cells induces MED1 phosphorylation at threonine T1457, which enables MED1’s physical interaction with AR at SEs. We assessed the interaction between AR and phosphorylated MED1 in clinical prostate tumor specimens (57 cases) using proximity ligation assays (PLA), and revealed that it occurred in CRPC specimens, and not in benign prostate tissues or muscles (Figure 5C and Supplementary Figure S5E). Thus, the interaction between phosphorylated MED1 and AR appears to be important in PCa cells and likely contribute to the induction of AR transcriptional activity. Therefore, we tested colocalization of AR foci with phosphorylated MED1 (pMED1, at T1457) and phosphorylated Pol II (pPol II, at S2). Using phospho-specific antibodies, we observed that a significant number of the AR containing foci colocalized with pMED1 and pPol II in LNCaP cells overexpressing AR-mEGFP (Figure 5B) or stained for endogenous AR (Supplementary Figure S5B). To further validate the findings, we performed PLA. These assays demonstrated interactions between AR, pMED1 and pPol II, respectively, as well as colocalization of interaction sites with AR-mEGFP containing foci (Figure 5D, Supplementary Figure S5F). These interactions were significantly reduced by 1,6-HD treatment (Figure 5E). Overall, our experiments confirm that AR, pMED1 and pPol II interact, and that they are co-present in many, but not all, AR foci in LNCaP cells.

To test the ability of AR to compartmentalize partners into its droplets *in vitro*, we expressed and purified the IDR of MED1 with an N-terminus OFPSpark red tag and mixed it with AR-mEGFP-MBP (Figure 5F) and His-AR-mEGFP (Supplementary Figure S5G). Confocal microscopy revealed that MED1-IDR colocalizes with AR droplets formed *in vitro*. Moreover, the number of AR droplets significantly increased in the presence of MED1-IDR (Figure 5F and Supplementary Figure S5H). Importantly, while AR does not form droplets below 0.03 μM, the addition of MED1 induces AR droplets that are significant in number and size (Figure 5F and Supplementary Figure S5H). These data suggest that the threshold concentration for AR phase separation could be lowered in presence of transcriptional activator such as MED1.

Next, we tested whether altering foci formation chemically or by cofactor silencing impacts AR transcriptional activity. Our PCa model and *in vitro* experiments revealed that AR foci and droplets are reduced in number at elevated 1,6-HD concentrations (Figures 2D and 3D). Moreover, previously described transcriptional condensates (14,16) formed by the coactivator MED1 in cells are also sensitive to 1,6-HD. Therefore, we expected 1,6-HD to affect the transcriptional activity of AR. Indeed, mRNA levels of AR-targeted genes *KLK3*, *FKBP5* and *TMPRSS2* were significantly reduced upon 1,6-HD treatment without any significant off-target effect on non-AR regulated genes *FKBP52*, *FXN*, and *HPRT* (Figure 5G and Supplementary Figure S5I). Given the importance of MED1 in the transcriptional activity of AR in PCa cells, we posited that MED1 repression may reduce foci formation and transcriptional activity of AR. Knocking down the expression of MED1 with siRNA, indeed reduced AR-rich foci formation significantly (Figure 5H). The effect of MED1 silencing on foci formation was mirrored by a lower AR transcriptional activity as seen by the reduced mRNA levels of AR-targeted genes *KLK3*, *FKBP5* and *TMPRSS2* (Figure 5I), genes that have been found within 100 kb of SE peaks in PCa cells (7). Consistent with these findings and the importance of MED1 phosphorylation for AR transcriptional activity, inhibition of MED1 phosphorylation by the CDK7 inhibitor THZ1 also reduced foci formation (Figure 5J). These experiments demonstrate that AR transcriptional activity and foci presence are correlated in androgen-dependent PCa cells and that both are dependent on MED1 expression and phosphorylation.

### Recruitment of MED1 but not AR to SE is sensitive to 1,6-hexanediol treatment

**Figure 6.**
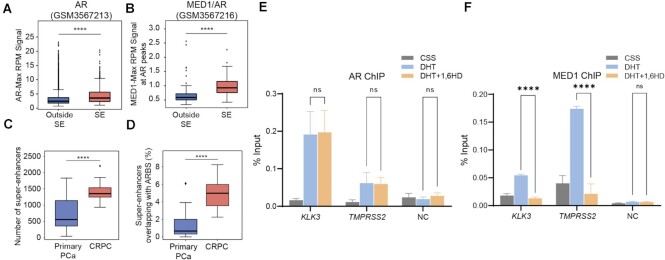
1,6 Hexanediol treatment affects MED1 recruitment to AR binding sites at SEs. (**A, B**) Binding intensities at AR binding sites (ARBS) within superenhancers (SE) was increased compared to non-SE sites for both AR (A) and MED1 (B). (**C**) Number of SEs in primary PCa and CRPC patient samples. SEs were called with the ROSE algorithm from published H3K27ac (GSE130408) of clinical samples from primary PCa or CRPC. (**D**) The percentage of AR binding sites (ARBS) localizing at SEs in CRPC compared to primary PCa. (**E, F**) Effect of 1,6-hexanediol (1,6HD) on AR and MED1 binding to specific SEs. LNCaP cells were hormone starved in 5% CSS for 72 h then treated with 1 nM DHT or ETOH for 2 h and followed by 2.5% 1,6-HD for 30 min. The crosslinked chromatin was incubated with AR (E) or MED1 (F) specific antibodies. Enrichment of AR and MED1 at *KLK3* and *TMPRSS2* sites as well as a negative control (NC) site were quantified by qPCR using specific primers. *P* values are indicated by stars: ns ≥ 0.05, * 0.01 to 0.05, ** 0.001 to 0.01, *** 0.0001 to 0.001, **** < 0.0001.

Transcriptionally active SEs in PCa VCap cells were found enriched in AR (together with MED1) upon dihydrotestosterone (DHT) stimulation compared to enhancer regions (7). We observed significant enrichment of both AR and MED1 occupancy at AR binding sites found in SEs compared to normal enhancers in LNCaP cells (Figure 6A, B), thus confirming that AR/MED1 enrichment at SE was not a cell-line specific phenotype. To determine if a similar enrichment for AR at SEs could be observed in clinical PCa samples we identified potential SEs from published H3K27Ac ChIPseq of primary PCa and CRPC with the ROSE algorithm (48,49) (GSE130408). The data confirmed an increase of SEs with the advancement of PCa (Figure 6C). Overlaying the identified SEs with high confidence clinical AR-binding sites (ARBS) (50) revealed a marked increase of AR-occupied SEs in CRPC as compared to primary PCa (Figure 6D).

We next tested whether recruitment of AR to SE sites was sensitive to 1,6-HD, a treatment that we showed to affect AR condensate/foci formation and transcriptional activity. To do so, we conducted chromatin immunoprecipitation (ChIP) of AR in presence and absence of 1,6-HD. We observed a strong enrichment of AR binding following androgen stimulation at the *KLK3* and *TMPRSS2* SEs (Figure 6E and Supplementary Figure S6). Interestingly though, 1,6-HD treatment did not affect AR binding to chromatin. We repeated these experiments with MED1 and also found an enrichment at the *KLK3* and *TMPRSS2* SE upon androgen stimulation (Figure 6F). However, 1,6-HD treatment significantly reduced MED1 binding to these AR SEs to a near basal level. Overall, these findings suggest that condensate formation may be important for the recruitment of transcriptional coactivators such as MED1 to SE sites subsequent to AR’s DNA binding.

## DISCUSSION

Coactivators such as BRD4 and MED1 have been shown to form nuclear foci at SEs that exhibit properties of liquid-like condensates. Moreover, the C-terminal IDR of MED1 forms phase-separated droplets that concentrate the transcriptional machinery from nuclear extracts. Here, we show that AR, a known interactor of BRD4 and MED1 (4,51), also forms nuclear foci upon specific stimulation with androgens in AR positive PCa cells. AR foci exhibit properties associated with liquid-like condensates such as responsiveness to 1,6-HD treatment and recovery of fluorescence after photobleaching. We also confirmed that recombinant full-length AR protein forms liquid-like droplets *in vitro*. Droplet formation *in vitro* is inhibited by high salt concentration and exposure to 1,6-HD, which suggests that both electrostatic and hydrophobic interactions are key for their formation. Importantly, FRAP experiments reveal similar diffusive exchange properties for *in vitro* droplets and foci formed by AR in PCa cells upon androgen stimulation. Moreover, recovery half-lives calculated from these FRAP experiments are similar to the ones measured for MED1 foci (Supplementary Figure S2B).

Different AR truncation constructs and domains have been shown recently to form droplets *in vitro* (17–19). However, we found that truncated AR constructs lacking the NTD, the DBD or the LBD are not able to form foci in cells despite being expressed to similar levels as full-length AR and being able to translocate to the nucleus. Taken together, these results suggest that multiple domains are required to drive foci formation in the environment of the PCa cells. Consistent with this conclusion, small molecule inhibitors of the LBD and NTD reduced foci formation of full-length AR in PCa cells, and the same inhibitors dissolved droplets formed by full-length AR *in vitro*. Moreover, recombinant full-length AR was able to form droplets *in vitro* at much lower concentrations than isolated domains (17–19), Overall, our findings suggest that AR foci could be phase-separated condensates that depend in their formation on AR’s multi-valency, a characteristic feature of many phase separation scaffolds including other transcription factors such as GR (31).

Our results also reveal that AR foci are sites of the transcriptional activity. AR foci co-localize with SE-associated gene *FKBP5* and with newly synthesized RNA in LNCaP cells as shown with DNA_FISH and immunofluorescence. A significant fraction of AR foci colocalizes with phosphorylated MED1 and RNA Pol II as seen by immunofluorescence and PLA assays. Moreover, reduced expression of one of the biomolecular partners of AR in these hubs, MED1, lowered the number of foci and AR transcriptional activity to a similar extend. Our findings are of particular interest in light of the recent study by Rasool *et al.* demonstrating that phosphorylation of MED1 at T1457 upon androgen stimulation is essential for AR-mediated transcription in PCa cells, i.e. the transcription of AR targets genes (e.g. *KLK3*, *TMPRSS2*) and oncogenic drivers (e.g. *ERG* and *MYC)* (7). The direct interaction between the AR and ERG oncogene has recently been confirmed by a report of the cryo-EM structure elucidating this interaction (52). Therefore, it will be interesting to unveil the role of ERG in AR condensate formation in future studies. Rasool *et al.* also revealed that MED1 phosphorylation can be impeded by a CDK7-specific inhibitor, THZ1, which blocks genome-wide co-recruitment of AR and MED1 (7). Consistent with this result, we find that THZ1 reduces AR foci formation. These findings suggest that androgen-induced AR foci are transcriptional hubs in AR positive PCa cells and their assembly depends on phosphorylated MED1.

**Figure 7.**
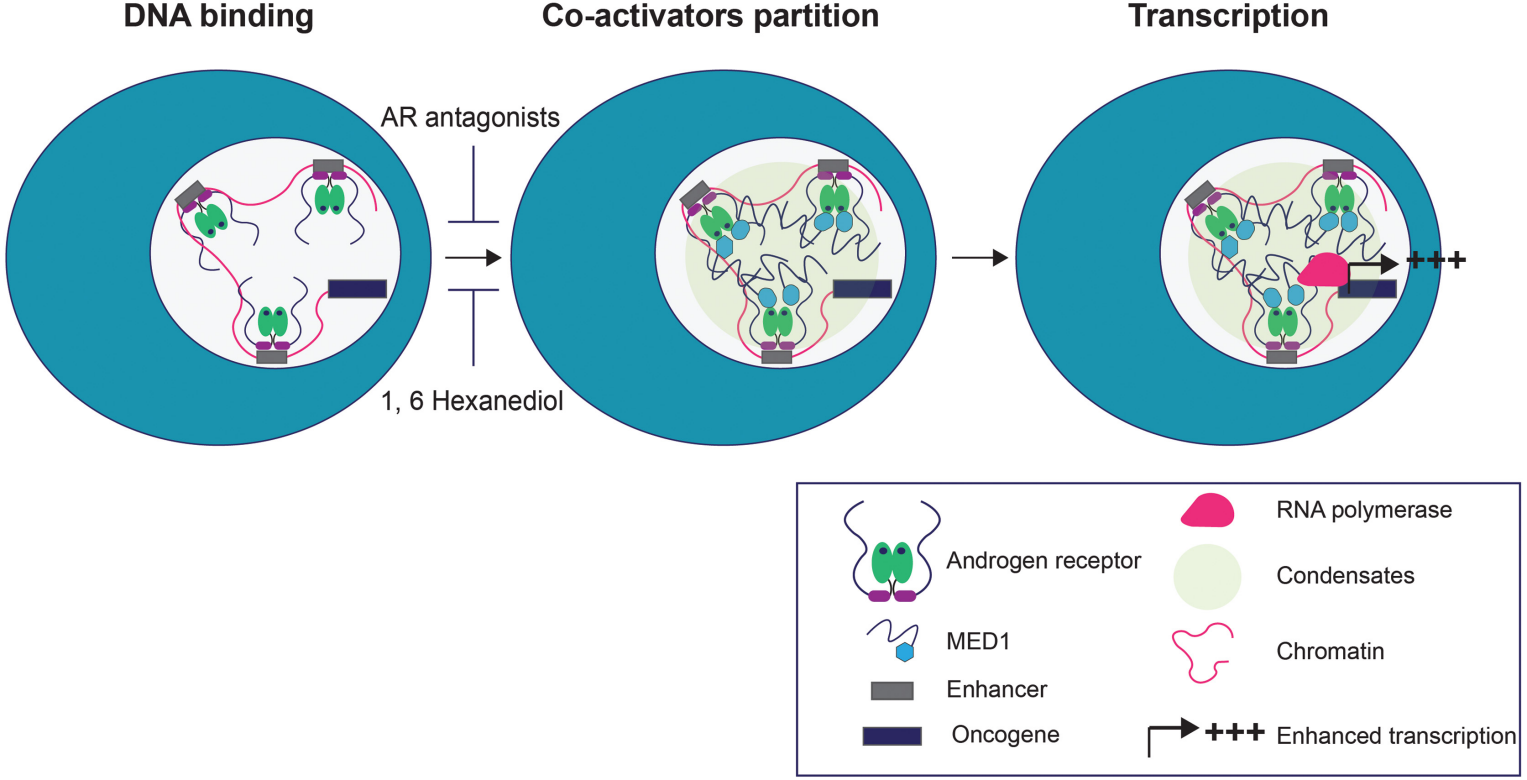
Model of the recruitment of the AR to superenhancers and the subsequent formation of transcriptional condensates. Our results suggest a model in which AR is recuited post-DHT stimulation to AREs at SEs, enabled by its DBD. Combinations of homotypic as well as heterotypic interactions, including interactions with coactivators such as MED1, may induced phase separatioin and the cooperative recruitment of other factors necessary for enhanced transcriptional activity in PCa cells. Antagonists and 1,6-hexanediol targeting homo and heterotypic AR interactions are preventing phase separation and thus foci formation by AR at SEs.

Under physiological conditions, SE programs control genes involved in cell identity. In cancer cells, by contrast, oncogenes acquire SE in order to assemble the transcriptional apparatus at high density and enable enhanced transcription of target genes (11,14,53). Mechanisms controlling the SE acquisition by oncogenic drivers include enhancer amplification, enhancer hijacking, and small insertions that create master transcription binding site (11). Dependence on SE programs has been reported for many cancers, including PCa (7,10,54). The majority of advanced PCas, including CRPC, are addicted to AR signalling and drive on SE-dependent oncogenic programs such as *TMPRSS2-ERG* and *MYC* (7). Consistent with this current understanding of PCa biology, we found an increase of SEs and AR-occupied SEs with the advancement of PCa in patients. Given that MED1 and AR interact not only in androgen-dependent PCa cells at SEs (7) but also in PCa tissues and that both these proteins are able to phase separate and form foci (14–16), it is likely that the partioning of AR and MED1, and other coactivators, to SEs in PCa is, at least in part, mediated by phase separation. Consistent with this idea, we found that the IDR of MED1 co-localizes to droplets formed by full-length AR *in vitro*. Moreover, 1,6-HD treatment significantly reduced mRNA levels of two AR target genes located within 100kb of SE peaks in PCa cells. Interestingly, 1,6-HD disrupted MED1 recruitment to AR DNA binding sites, but did not affect AR binding to its target gene. These results point toward a model where AR may be partitioned to coactivator condensates downstream of its SE binding in PCa cells (Figure 7). Consistent with this model, inhibition of DNA binding with VPC-14449 reduces foci formation in cells and ROX-ARE recruitment to condensates *in vitro* but has no effect on AR phase separation. By contrast the NTD (EPI-001) and LBD inhibitors (bicalutamide and enzalutamide), which impede homotypic (N/C) and heterotypic (coactivator) interactions, reduce foci formation in PCa cells and condensate formation *in vitro* (39,40). Additionally mutations in the AF2 site of the LBD—important for both N/C interactions and coactivator specific recruitment—or in the DNA binding pocket of the AR reduced the receptor ability to form foci, validating the essential role of each individual domain in enabling AR foci formation in the cell.

In summary, we reveal that dynamically formed foci are important to the transcriptional activity of AR in PCa cells. This transcriptional activity can be modulated by changing the foci content genetically or chemically. A better understanding of foci assembly mechanism and their mode of action may open novel therapeutic venues to treat advanced forms of PCa, specifically those that are addicted to androgen-driven SE transcriptional programs.

## DATA AVAILABILITY

The data that support the findings of this study are available from the corresponding author, upon reasonable request.

## Supplementary Material

gkac1158_Supplemental_Files
