# Supplementary material for: Dynamic phase separation of the androgen receptor and its coactivators key to regulate gene expression
Source: Nucleic Acids Res. 2022 Dec 20;51(1):99–116. doi: 10.1093/nar/gkac1158 (PMC9841400; doi:10.1093/nar/gkac1158)
Supplement: gkac1158_Supplemental_Files [file gkac1158_supplemental_files.zip › Lallous_MovieS1_Legend.pdf]

Movie S1: Time-lapsed AR foci formation in LNCaP cells upon DHT stimulation. Cells grown in glass-bottom dish were transfected with AR-mEGFP and cultured in 5% CSS media for 2 days. The dish was then moved to the incubator on top of the confocal microscope and maintained in 5% CO_2 with humidity at 37°C. Cells were then stimulated with 1nM DHT and the images were taken with the z-stack model.
