# Supplementary material for: Dynamic phase separation of the androgen receptor and its coactivators key to regulate gene expression
Source: Nucleic Acids Res. 2022 Dec 20;51(1):99–116. doi: 10.1093/nar/gkac1158 (PMC9841400; doi:10.1093/nar/gkac1158)
Supplement: gkac1158_Supplemental_Files [file gkac1158_supplemental_files.zip › Lallous_Supplementary_information_Proof.pdf]

Dynamic phase separation of the androgen receptor and its coactivators key to regulate gene expression.

Fan Zhang[1], Maitree Biswas[1$], Shabnam Massah[1$], Joseph Lee[1], Shreyas Lingadahalli[1], Samantha Wong[1], Christopher Wells[1], Jane Foo[1], Nabeel Khan[1], Helene Morin[1], Neetu Saxena[1], Sonia H. Y. Kung[1], Bei Sun[1], Ana Karla Parra Nuñez[1], Christophe Sanchez[1], Novia Chan[1], Lauren Ung[1], Umut Berkay Altıntaş[2,3], Jennifer M. Bui[4], Yuzhuo Wang[1], Ladan Fazli[1], Htoo Zarni Oo[1], Paul S. Rennie[1], Nathan A. Lack[1,2,3], Artem Cherkasov[1], Martin E. Gleave[1], Jörg Gsponer[4]* and Nada Lallous[1]*.

[1] Vancouver Prostate Centre, Department of Urologic Sciences, University of British Columbia, 2660 Oak St., Vancouver, BC, V6H 3Z6, Canada
[2] School of Medicine, Koç University, Rumelifeneri Yolu, Istanbul, 34450, Turkey.
[3] Koç University Research Centre for Translational Medicine (KUTTAM), Koç University, Rumelifeneri Yolu, Istanbul, 34450, Turkey.
[4] Michael Smith Laboratories, Department of Biochemistry and Molecular Biology, University of British Columbia, Vancouver, BC, V6T 1Z4, Canada.

* To whom correspondence should be addressed. Tel: +16048754111; Fax: +1604-875-5654; Email: nada.lallous@ubc.ca. Correspondence may also be addressed to gsponer@msl.ubc.ca.

[$] Authors contributed equally to this work.

Supplementary data:

Movie S1: Time-lapsed AR foci formation in LNCaP cells upon DHT stimulation. Cells grown in glass-bottom dish were transfected with AR-mEGFP and cultured in 5% CSS media for 2 days. The dish was then moved to the incubator on top of the confocal microscope and maintained in 5% CO_2 with humidity at 37°C. Cells were then stimulated with 1 nM DHT and the images were taken with the z-stack model.

Supplementary figures:

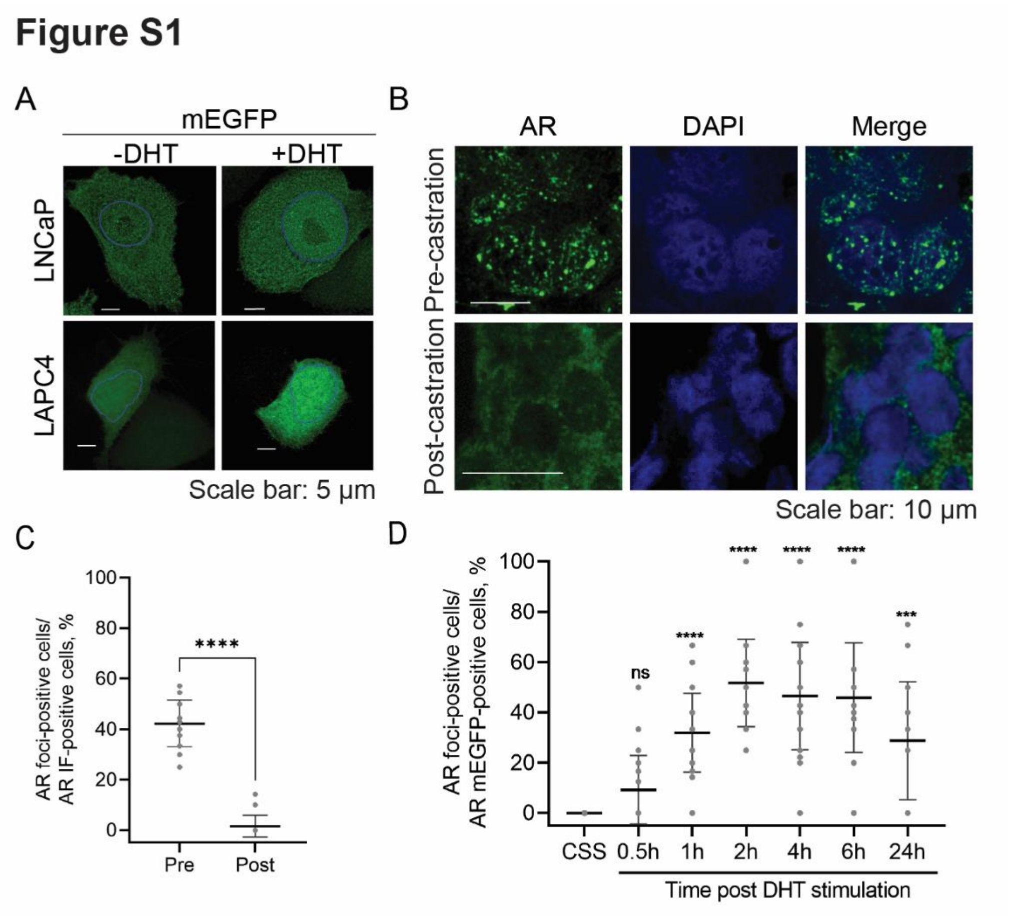

Figure S1: AR-rich foci form upon androgen stimulation. A- The vector encoding mEGFP protein doesn't form foci upon androgen stimulation. mEGFP-transfected cells were starved in 5% CSS containing medium for two days and then stimulated with 1nM of DHT for 2 hours. The localization and distribution of mEGFP protein was inspected under confocal microscope. Nucleus was outlined in blue. B- The patient derived xenograft (PDX) PCa tumor lines AB313H, AB310F and AB676-2 were grafted to mice supplemented with testosterone (10 mg/mouse). Mice in the treatment arm received castration for one week. OCT blocks of tumor tissue were prepared and 5 µm thick tissues were sectioned. IF was performed to visualize the AR. The images were taken with the confocal microscope using the Z-stack model. C- Percentage of AR foci-positive cells over AR IF positive cells were quantified from 5 fields of each sample (N = 3). D- LNCaP cells expressing AR-mEGFP were starved for 2 days in 5% CSS and then stimulated with DHT (1 nM) for various time as indicated. The percentages of foci-containing cells against the AR-mEGFP positive cells were presented with mean ±

SD. A total of 45 fields were evaluated from 3 biological replicates (N=3). p values are indicated by stars: ns ⩾ 0.05, * 0.01 to 0.05, ** 0.001 to 0.01, *** 0.0001 to 0.001, **** < 0.0001.

Figure S2

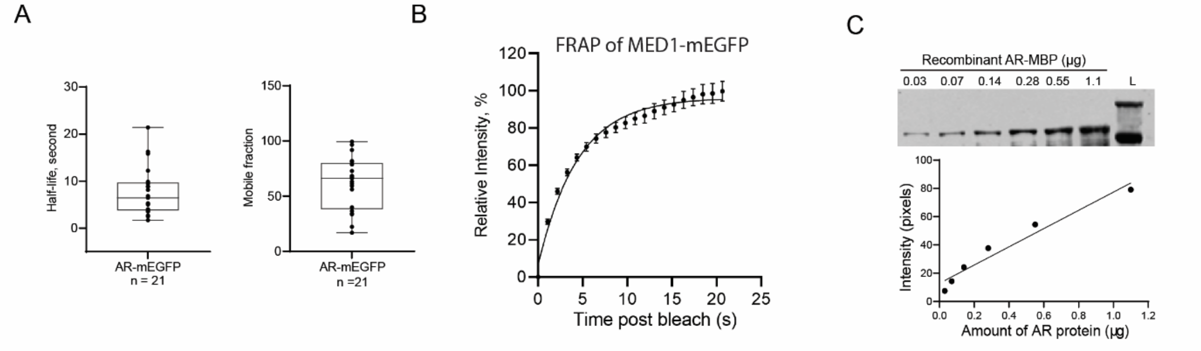

Figure S2: Characteristics of AR-rich condensates. A- FRAP quantification of half-life and mobile fraction. B- FRAP assay to examine the diffusion of MED1 in and out the condensates. LNCaP cells transfected with MED1-mEGFP were cultured in 5% CSS for 2 days and then stimulated with 1 nM DHT for 2h. MED1-mEGFP foci were photobleached and the fluorescence recovery was monitored for 30 seconds. The relative intensity was presented as mean ± SD. A total of 24 cells were analyzed from 3 biological replicates. C- Titration curve of AR by using a purified recombinant AR-MBP-His protein.

Figure S3

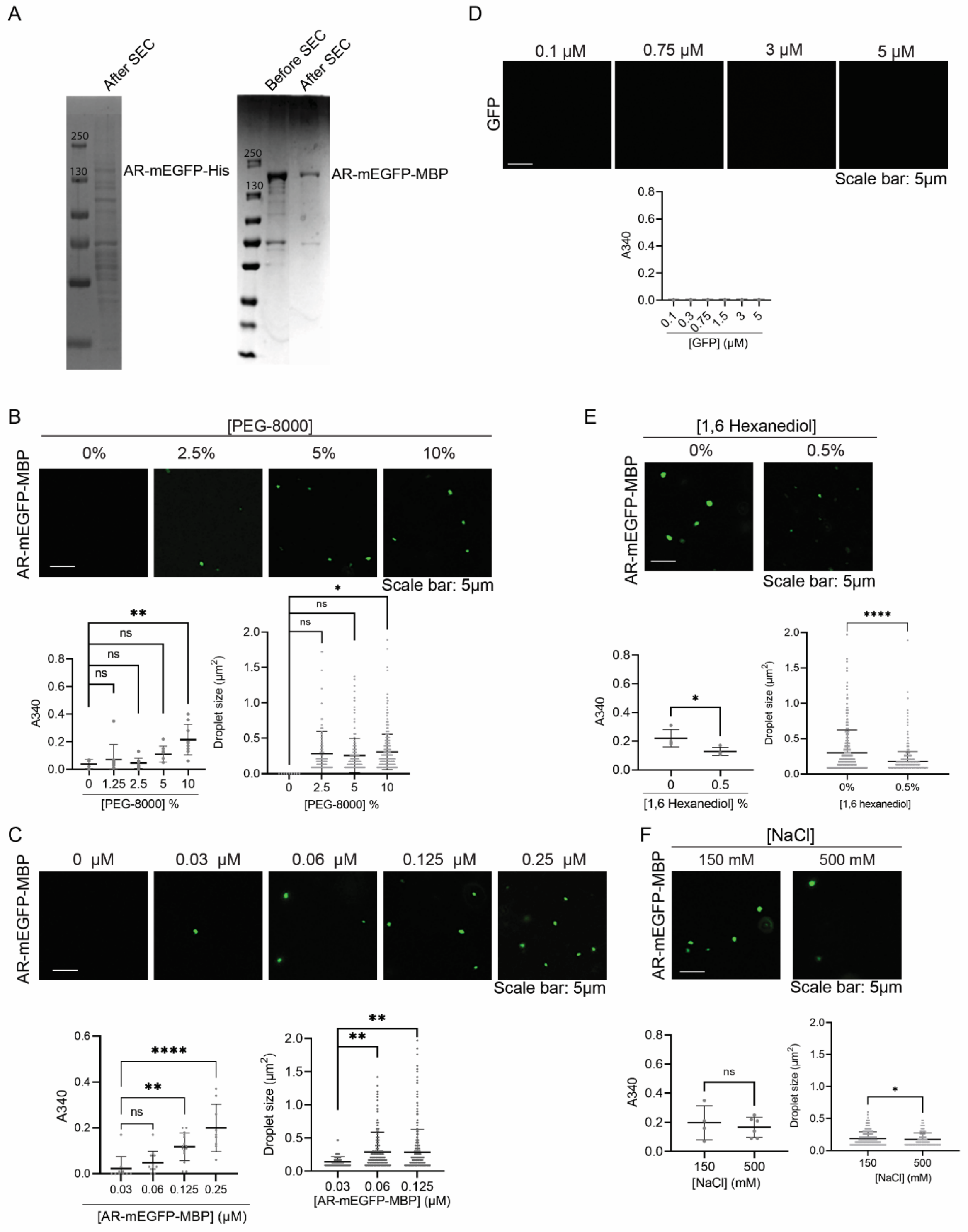

Figure S3: Characteristics of AR-mEGFP-MBP protein *in vitro* condensates. A- Comparison of sample purity between His-AR-mEGFP and AR-mEGFP-MBP proteins as seen on SDS PAGE. B- Effect of PEG-8000 concentration on AR-mEGFP-MBP droplet formation and their sizes as characterized by confocal microscopy and turbidity assay that measures the increase in light scattering upon droplet formation by following the optical density at 340 nm. C- AR droplets formation is dependent on protein concentration (10% PEG-8000 and 20 minutes incubation time). D- Recombinant GFP protein is used as control. Effect of 1,6 hexanediol (E) and salt concentration (F)

on AR droplet formation and their sizes. Protein concentration is 0.125 µM. Scale bar: 5µm. p values are indicated by stars: ns ⩾ 0.05, * 0.01 to 0.05, ** 0.001 to 0.01, *** 0.0001 to 0.001, **** < 0.0001.

Figure S4

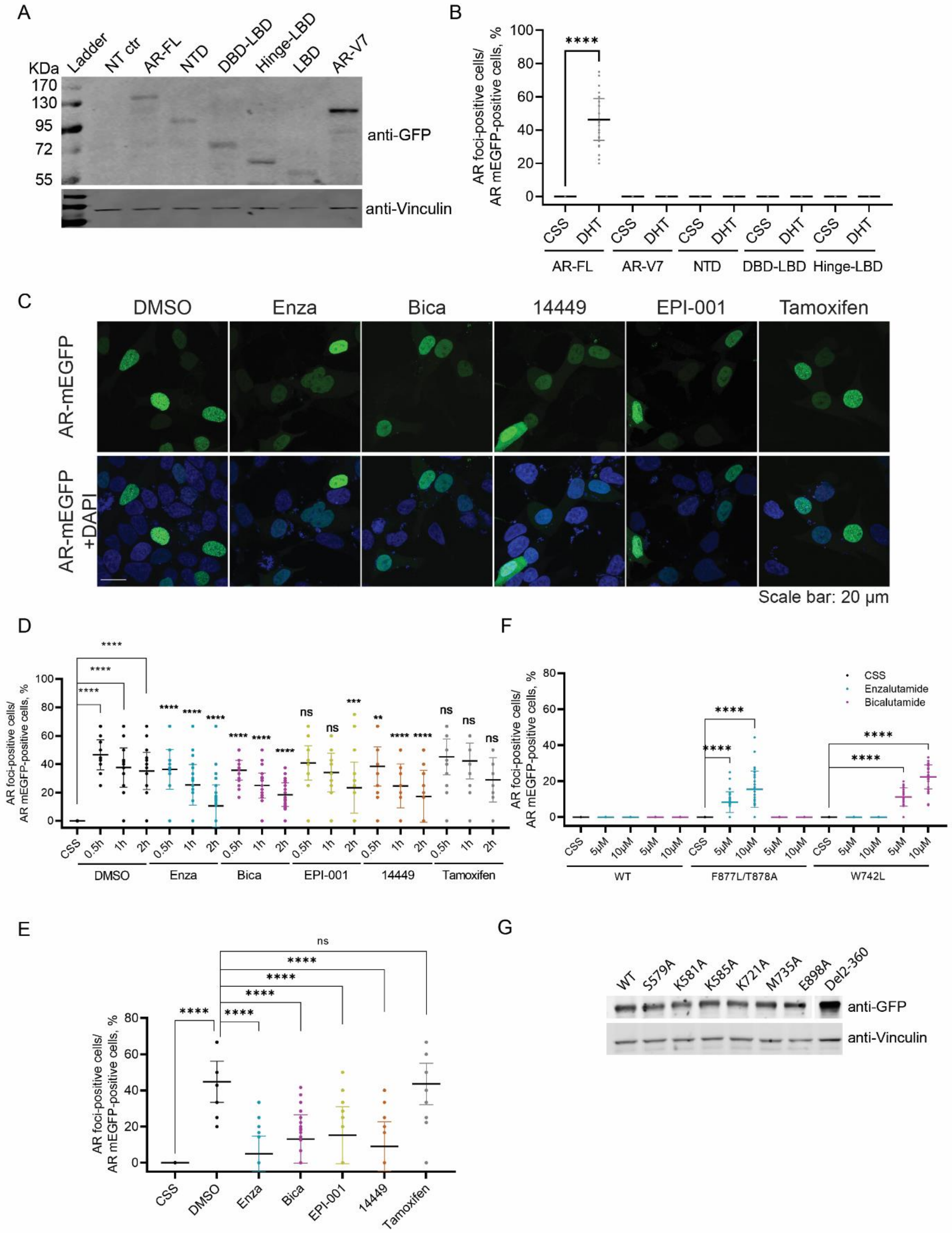

Figure S4: Full Length AR is required for foci formation. A- LNCaP cells transfected with truncated forms of AR-mEGFP were cultured in 5% CSS for 2 days and then stimulated with 1 nM DHT for 2h. Protein lysates were collected and proceeded for Western blot to monitor the expression levels of mEGFP-tagged AR truncations. AR-FL: AR full length, AR-V7: AR variant protein V7 , NTD: N-terminal domain, DBD: DNA-binding domain, LBD: ligand-binding domain, Hinge: hinge region between DBD and LBD. B- Quantification of foci formation in LNCaP cells transiently transfected with the truncated AR forms upon starvation for 2 days and then stimation with 1nM DHT for 2h. The aligned dotted blot presents the data from 45 fields with mean ± SD from three independent experiments. two-tailed unpaired Student's t-test was used to analyze the data. C-D AR-mEGFP transfected LNCaP cells were starved for 2 days in CSS and then were treated with 1 nM DHT for 2h followed by treatment with the compounds (10 μM) for 30 min, 1h and 2h. Cells were fixed and the images were taken with a confocal microscope. C- Images corresponding to 2h post treatements. D- Quantification of the impact of AR antagonists on AR-mEGFP foci formation in LNCaP cells. The aligned dotted blot presents the data from 45 fields and from three independent experiments with mean ± SD. E- AR-mEGFP transfected LNCaP cells starved for 2 days, were pre-treated with the compounds for 2 hours prior to the DHT stimulation of 2h. Percentage of foci containing cells were quantified from 45 fields of three independent experiments. The data is presented as overlapping dotted blot with mean ±SD. F- Plasmids expressing mEGFP-tagged wild type AR (WT) or either F877L/T878A or W742L AR mutants were transfected into LNCaP cells. Cells were starved with 5% CSS for two days and then increasing concentrations of bicalutamide or enzalutamide were added for 2h without DHT stimulation. AR foci formation was then quantified. The aligned dotted blot presents the data from 45 fields and from three independent experiments with mean ± SD. G- LNCaP cells were transfected with the indicated AR mutant plasmids and starved in 5% CSS for 2 days. Cells were then stimulated with 1 nM DHT for 2h and protein lysates were collected and proceeded for western blot to monitor the protein levels of the mutants (GFP). Vinculin was used as a loading control.

Figure S5

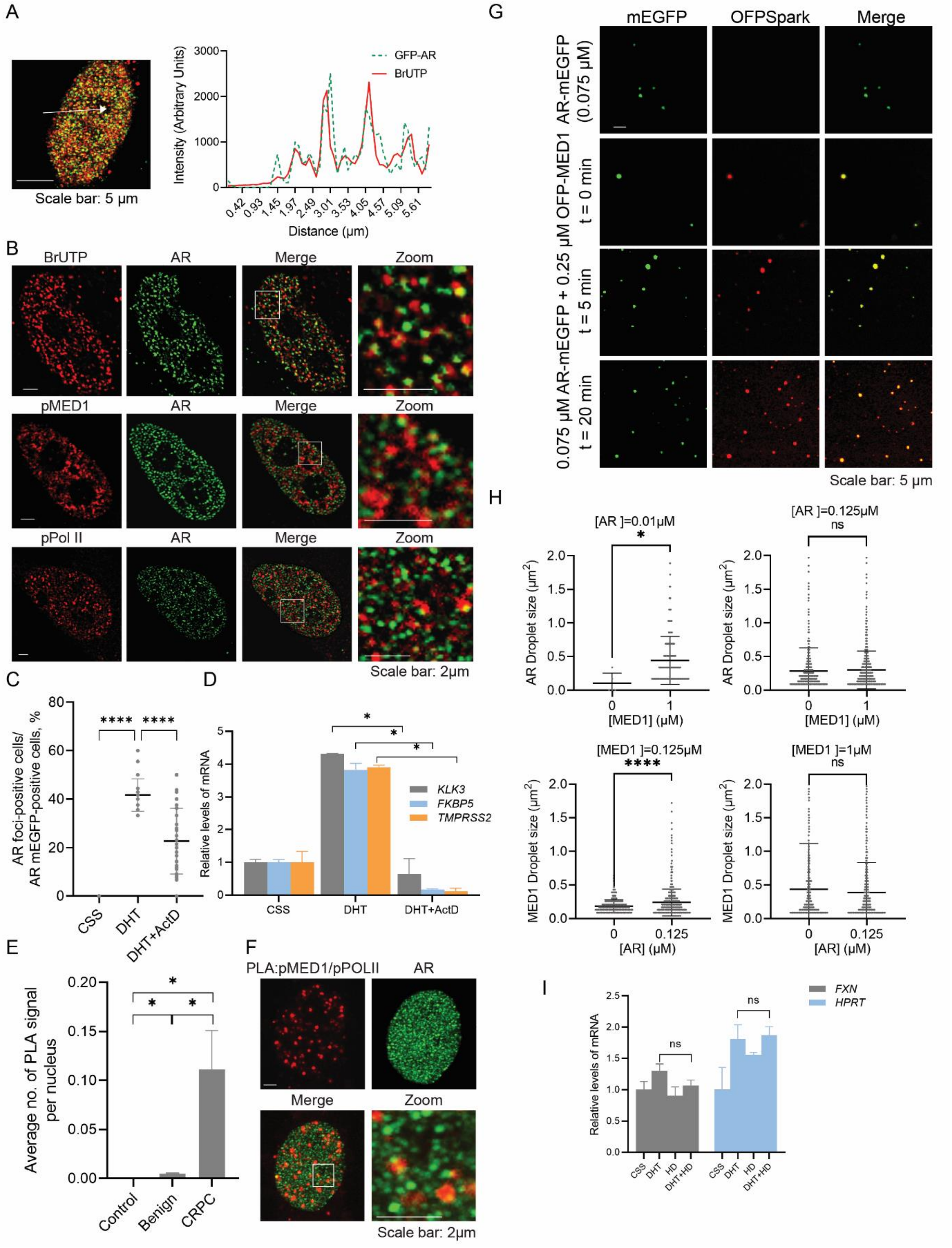

Figure S5: Foci colocalize with AR transcriptional machinery. A- Colocalization of AR foci (green) with nascent RNA as detected by BrUTP IF(red). Intensity quantification (right panel) in red and green channels across the white arrow shown in the left panel. B- LNCaP cells starved with 5% CSS for two days were stimulated with DHT for 2 hours. IF on BrUTP, pMED1 and pPol II (red panels) and endogenous AR (green) were performed. The co-localization with AR-rich condensates was

examined under confocal microscope. C- LNCaP cells transfected with AR-mEGFP were grown in 5% CSS for two days. Cells were then treated with 1 µg/ml of Actinomycin D (ActD) for 4 hours and stimulated with 1 nM DHT for 2 hours. The foci formation was quantified from 45 fields and from three independent experiments and the data was presented in aligned dotted blot with mean ± SD. D- LNCaP cells grown in 5% CSS for 3 days were pre-treated with or without 1 µg/ml of ActD for 4 hours and then stimulated with 1 nM DHT for 8 hours. Total RNA was extracted and proceeded for qRT-PCR on the indicated genes. E- Quantification of *in-situ* tissue proximity ligation assay (PLA) assay of AR and phospho-MED1 in control (n = 2; muscle and brain), benign prostate (n = 3) and CRPC tissues (n = 8). (mean ± SEM; two-tailed t-test). F- Combined PLA staining (in red, mouse anti-pPOLII and rabbit anti-pMED1) and IF staining of endogenous AR (green panel, goat anti-AR) were carried out in LNCaP cells starved in 5%CSS then stimulated with 1 nM DHT. The colocalization of PLA signal with AR-rich condensates was inspected under the confocal microscope. Images in the white frames were enlarged and displayed in the right panel. G- His-AR-mEGFP *in vitro* droplets incorporate MED1-IDR. H- Quantification of the changes in size of AR and MED1 droplets when the two proteins are mixed together *in vitro*. Data are presented as scatterd points with mean ± SD (n=5). I- Effect of 1,6-hexanediol (HD) on mRNA expression. Cells were starved for 3 days in CSS and then treated with 1nM DHT ± 2.5% HD for 30 min, and then washed and incubated with DHT containing medium for 16 hours. Total RNAs were extracted and the mRNA levels of genes of interest were examined using qRT-PCR. Values are expressed as mean ± SD. p values are indicated by stars: ns ⩾ 0.05, * 0.01 to 0.05, ** 0.001 to 0.01, *** 0.0001 to 0.001, **** < 0.0001.

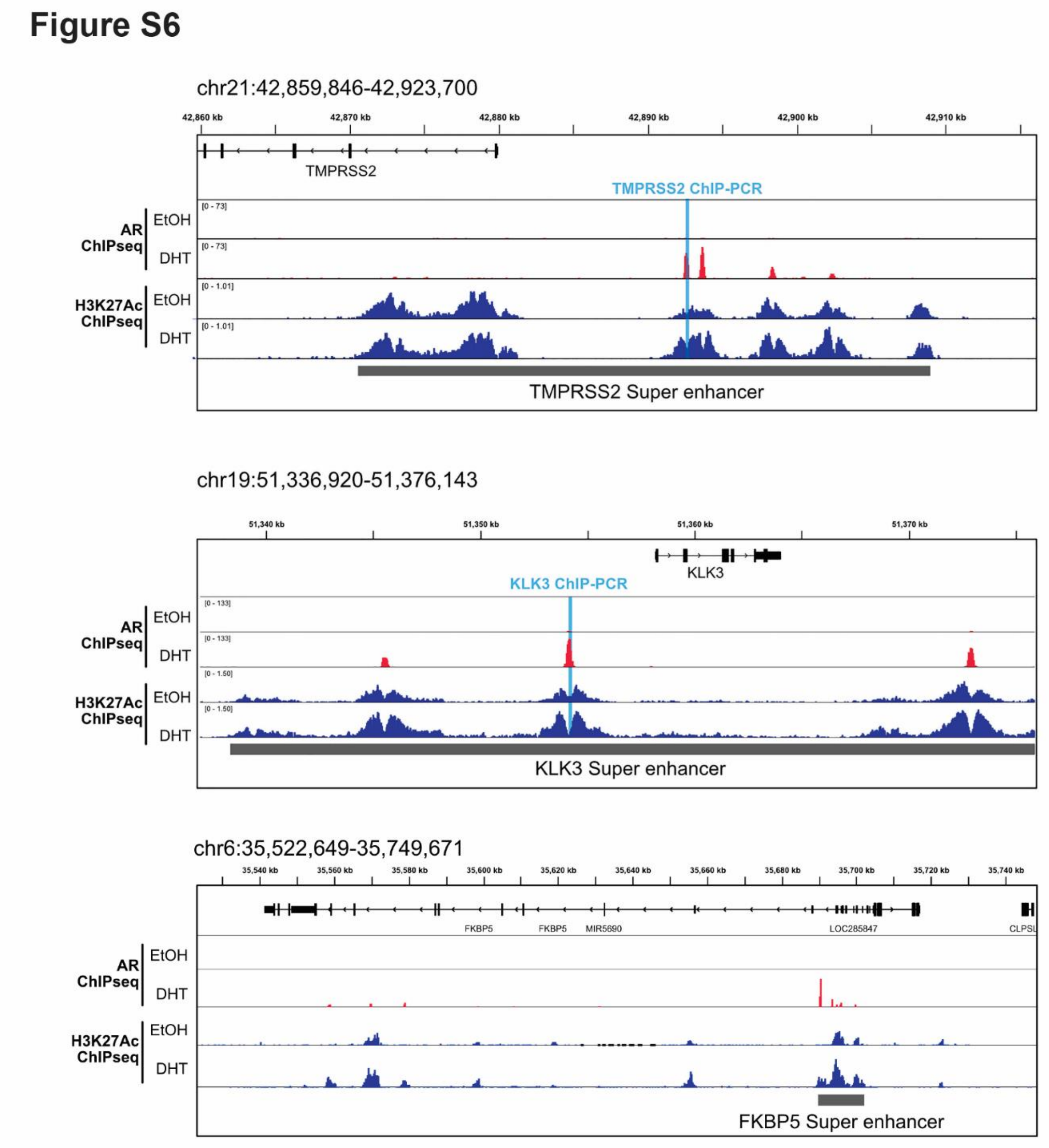

Figure S6: Superenhancers of *TMPRSS2*, *KLK3*, and *FKBP5* genes in LNCaP cells. AR and H3K27Ac signals from ChIP-seq data. AR (red; GSE83860) and H3K27Ac (blue; GSE130408) ChIPseq in LNCaP cells were overlaid with SE called with the ROSE algorithm. The primers used in ChIP-qPCR are shown in light blue (*KLK3* and *TMPRSS2*). No ChIP-PCR was done with the *FKBP5* binding site.
